# Specific binding between *Arabidopsis thaliana* phytochrome-interacting factor 3 (AtPIF3) bHLH and G-box originated prior to embryophyte emergence

**DOI:** 10.1186/s12870-024-05777-z

**Published:** 2024-11-11

**Authors:** Kuan-Ting Hsin, Yu-Hsuan Lee, Kai-Chun Lin, Wei Chen, Yi-Sheng Cheng

**Affiliations:** 1 Department of Tropical Agriculture and International Cooperation, National Pingtung University of Science and Technology, Pingtung, 912301 Taiwan; 2 Department of Life Science, College of Life Science, National Taiwan University, Taipei, 106319 Taiwan; 3 Institute of Plant Biology, College of Life Science, National Taiwan University, Taipei, 106319 Taiwan; 4 Genome and Systems Biology Degree Program, College of Life Science, National Taiwan University, Taipei, 106319 Taiwan

**Keywords:** Circadian clock, DNA binding preference, Conservation, Protein-DNA interaction

## Abstract

The basic helix-loop-helix (bHLH) domain via critical amino acid residues on basic region binding to E-box (5′-CANNTG-3′) is known in embryophyte. However, the dictated E-box types selection by bHLH dimers and the significant impact of these critical amino acid residues along embryophyte evolution remain unclear. The *Arabidopsis thaliana* PIF3-bHLH (AtPIF3-bHLH) recombinant protein and a series of AtPIF3-bHLH mutants were synthesized and analyzed. The reduced DNA binding ability and affinity of AtPIF3-bHLH point-mutation proteins, observed via fluorescence-based electrophoretic mobility shift assay (fEMSA) and isothermal titration calorimetry (ITC), suggest the critical role of these DNA-recognition sites in maintaining the AtPIF3-bHLH–DNA interaction. The purifying selection signals and the DNA-recognition-site conservation at the species level suggest the invariant roles of these sites throughout embryophyte evolution. The G-box outcompeted other types of E-box for binding in our competitive fEMSAs. The dynamic hydrogen bond formed between AtPIF3-bHLH and the G-box core indicates flexible identification of the core region. These features highlight a fast fixation of the bHLH-G-box recognition mechanism through embryophyte evolution and serve as a blueprint for studying DNA recognition determinants of other TF families.

**Supplementary Information:**

The online version contains supplementary material available at 10.1186/s12870-024-05777-z.

## Introduction

The light changes that accompany diurnal rhythms are thought to be a signal that regulates land plant growth and development [1–4]. Phytochrome-interacting factor 3 (PIF3) interacts with the Pfr form of phytochrome B when responding to light stimuli [3, 5–8]. PIF3 is a well-characterized coordination node for converging photosensory signaling pathways and the circadian clock via protein–DNA recognition [4]. By binding to the promoter of its target genes, PIF3 oscillates with the regular periodicity of light/dark cycles, along with TIMING OF CAB EXPRESSION 1 (TOC1), PSEUDO-RESPONSE REGULATOR 3 (PRR3), PSEUDO-RESPONSE REGULATOR 7 (PRR7), and PSEUDO-RESPONSE REGULATOR 9 (PRR9). In *Arabidopsis*, PIF3 constitutively modulates expression of target genes under the control of the circadian clock [9]. Presumably, this mechanism maintains correct photosynthesis gene expression phasing in higher plants [1, 10]. In *Arabidopsis thaliana* and *Zea mays*, peak expression of most photosynthetic genes occurs around dawn [11, 12]. In crassulacean acid metabolism (CAM) plants, such as *Ananas comosus*, reprogramming the diel pattern of key metabolic genes was found to lead to high levels of CAM gene expression at night [13]. Given the functional significance of PIF3 and its orthologues in generating the rhythmicity of photosynthesis gene expression, investigating the evolution of PIFs in embryophytes would offer clues toward the evolutionary changes responsible for light-induced regulatory mechanisms.

In a prior study, examining putative PIF homologs found in the genome of the moss *Physcomitrella patens*, phylogenetic and functional analyses provided evidence for the evolutionary conservation of light-dependent PIF transcription activity in land plants [14, 15]. Recently, a genome-wide survey of 40 plant species revealed the presence of 246 PIFs across plant lineages, from bryophytes to core eudicots [15]. All identified PIFs harbor active phytochrome B binding (APB) and basic helix-loop-helix (bHLH) domains, suggesting conservation of these functional domains at the protein level. In addition, analyzing PIFs from diverse plant species revealed high sequence similarities among bHLH domains, hinting at its functional conservation [15]. At gene level, the selection pressure (ω = dN/dS) derived from calculating the ratio of non-synonymous substitution (dN) to synonymous substitution (dS) of PIF genes exhibited either positive selection (ω > 1) or purifying selection signal (ω < 1), suggesting high variation of nucleotide compositions of non-domain region comparing to domain regions. Compared to PIFs from other plant species, PIFs from members of the *Arabidopsis* PIF family, designated PIF1–8, are far better studied [3, 16–21]. Different sets of target genes harboring overlapping DNA-binding motifs are regulated by individual *Arabidopsis* PIFs [21].

The bHLH transcription factor (TF) family is the largest among plants [22]. The bHLH TFs have been found to have a broad range of key functions, including in stress responses, seed germination, organ differentiation, jasmonate synthesis-related regulation, cell elongation, and responses to environmental cues [23]. It is assumed that the DNA-recognition ability of bHLH plays a key role in its functions and allows bHLH TFs to participate in diverse molecular regulatory networks. The homo-tetramer form of MYC2-bHLH has been shown to enhance the binding ability of G-box (5′-CACGTG-3′) and the transcriptional regulation of target genes in *Arabidopsis thaliana* [24]. Each MYC2 homodimer can bind to its target DNA (5′-CACGTG-3′) with H453, E457, and R461 in the basic region, interacting with a half-site of the aforementioned DNA target and suggesting a symmetric DNA-binding form [24]. Asymmetric DNA-binding forms of bHLH have also been identified [25–30]. The PHO4 homodimer binds to E-box with an asymmetric bHLH–DNA form [30]. An asymmetric binding form was also identified in the CLOCK–BMAL1-bHLH heterodimer [29]. Regardless of whether bHLH is part of a homodimer or heterodimer, the bHLH–DNA binding form can therefore be either symmetric or asymmetric in form, and these two forms exhibit different nucleotide preferences for the G-box or E-box (5′-CANNTG-3′) [26, 28, 29].

A comprehensive survey of human bHLH binding preferences showed that bHLH TFs could be organized into three distinct clusters: cluster 1 comprises bHLH TFs that recognize a CAC half-site, cluster 2 shows CAT half-site preference, and cluster 3 TFs exhibit CAG half-site binding preference [31]. The amino acid residues located in the basic region that recognize DNA and the target DNA nucleotide compositions are therefore two key factors in determining bHLH–DNA binding mechanisms.

Here, we aimed to elucidate the roles of bHLH target (G- or E-box) nucleotide-recognition sites and the composition of core target nucleotide regions via evolutionary and structural analysis. We chose PIF3 because it is critical in mediating light-signal transduction and circadian-clock regulation in plants [4, 12, 20, 32–34]. To determine the degree of gene conservation, we first examined the evolutionary patterns (selection pressure change among selected PIFs) and sequence similarities (nucleotide diversity calculation by using sliding window method) among PIFs retrieved from representative embryophytes. Then, site-specific point mutations of DNA-contacting amino acid residues located in the basic region of bHLH were performed to assess any changes in binding affinity. Third, competition assays were performed to test the binding preference of artificially mutated nucleotides in the core region and of bHLH recombinant proteins.

## Materials and methods

### Reconstructing the phylogeny of PIFs identified from selected embryophytes

First, we retrieved the PIFs of *Arabidopsis thaliana* from the Arabidopsis Information Resource (TAIR, https://www.arabidopsis.org/). In total, eight AtPIFs were obtained. To reconstruct the relationship among C3, C4, and CAM plants, we selected three representatives: *Arabidopsis thaliana* (C3), *Z. mays* (C4), and *Ananas comosus* (CAM). The AtPIFs were then submitted to the online Phytozome database (https://phytozome.jgi.doe.gov/pz/portal.html#) [35] to identify orthologues in *Z. mays* and *Ananas comosus*. We also retrieved the AtPIF orthologues of *Marchantia polymorpha* and *Physcomitrium patens* for use as outgroups in our phylogeny reconstruction. During the BLAST process, only sequence similarity over 80% was kept. We thus identified 25 PIFs: one from *M. polymorpha*, three from *P. patens*, six from *Z. mays*, seven from *Ananas comosus*, and eight from *Arabidopsis thaliana*.

The PIF sequences were aligned using the MUSCLE [36] algorithm implemented in MEGA v.6 [37]. We then visually refined the resulting alignment matrix based on amino acid translations, using Bioedit [38]. The GTR + R model was selected as the best-fitting model for the PIF dataset based on the Bayesian information criterion (BIC) [39]. We used both BI and ML algorithms to infer the relationships among PIF genes, using the PhyML 3.0 online web interface [40]. The statistical support for nodes was assessed using the approximate likelihood ratio test (aLRT) and a Bayesian-like transformation of aLRT (aBayes), respectively [41, 42]. The conventional bootstrap algorithm was run with 1000 replicates.

### Molecular evolution of embryophyte clades

To evaluate the molecular evolution of *Arabidopsis thaliana*, *Z. mays*, and *Ananas comosus* PIFs, we tested the PIF sequences for changes in selective pressure (ω) after gene duplication and in their assigned clades by using CODEML, which is implemented in PAML v.4.0 [43]. To evaluate the change in selective pressure of a duplication event of interest or a specific clade, we used the branch model and branch–site model implemented in CODEML. The branch model considers changes in the value of ω among branches, while the branch–site model evaluates such changes among both branches and sites [43, 44]. Null and alternative models were generated for branch and branch–site models, respectively, and then compared. To verify the significance of the alternative and null models, we measured their likelihood values and tested them using the likelihood ratio test (LRT).

Due to the key role of AtPIF3 in governing *Arabidopsis thaliana* development, we assumed that clade C is under strong genetic constraint. Clade C was thus assigned as the foreground to test whether it was subject to strong purifying selection. Genetic constraints can also be released with a recent duplication event; to test this, we set up a pre- and post-duplication scenario [45]. Lastly, to examine the selective pressure among amino acid residues in our PIF dataset, we ran the site model implemented in CODEML.

### Examining nucleotide diversity along embryophyte PIFs using the sliding window method

To explore the changes in nucleotide diversity (π) in our PIF alignment, we used the sliding window method. The window was 30 nucleotides in width, and the step size was 10 nucleotides. The resulting π values were visualized using Excel.

### Identification of bHLH amino acid residue conservation via sequence logo

The bHLH region exhibited relatively low π values compared to other PIF regions analyzed in this study. To examine the sequence conservation of amino acid residues in the bHLH domain, we extracted the bHLH domain region and submitted it to WebLogo 3 (https://weblogo.threeplusone.com/) to determine the sequence conservation level across embryophytes [46].

### Protein expression and purification

To produce the PIF3-bHLH recombinant protein in *E*. *coli*, a DNA fragment containing the bHLH domain of the *PIF3* gene (AT1G09530) was amplified via polymerase chain reaction (PCR) using a designed primer set. This DNA fragment was then cloned between the *NheI* (5′-GCTAGC) site and the *XhoI* (3′-CTCGAG) site in the pET21b vector (Novagen, NJ, USA). The expression vector encoding C-terminal-His_6_-tagged truncated PIF3 was then transformed into the Rosetta (DE3) strain (Novagen). The cultures were grown at 37 °C to mid-log phase (OD_600nm_ ~ 0.375) followed by induction with 0.1 mM IPTG at 20 °C. After an overnight induction with IPTG, cells were harvested by centrifugation and stored at − 20 °C until required. To purify the PIF3-bHLH–His_6_ protein from the cell lysate solubilized in buffer A (30 mM HEPES, 500 mM NaCl, 20 mM Imidazole, 1 mM PMSF, pH 7.4), the supernatant was applied to a HisTrap FF column (Cytiva, Marlborough, MA, USA) pre-equilibrated with buffer B (30 mM HEPES, 500 mM NaCl, 20 mM Imidazole, pH 7.4). The column was washed with 10 column volumes of buffer B and then eluted with a linear gradient of buffer C (30 mM HEPES, 500 mM NaCl, 500 mM Imidazole, pH 7.4). The elution fractions were pooled and concentrated to the desired volume using an Amicon Ultra-15 centrifugal filter unit (3 kDa cut-off; Millipore, MA, USA). As a final polishing step, the concentrated protein sample was subjected to gel-filtration chromatography on a Superdex75 gel-filtration column pre-equilibrated with buffer D (30 mM HEPES, 500 mM NaCl, pH 7.4). The peak fractions were supplemented with glycerol (added to a final concentration of 10%) followed by concentration and storage at − 20 °C (Supplemental Fig. 1).

### Competitive fluorescence-based electrophoretic mobility shift assay (fEMSA)

In the analysis of protein–DNA interactions between PIF3-bHLH and an array of E-box sequences (Supplemental Table 1), the double-stranded DNA (dsDNA) probes used were designed according to G-box oligonucleotide probes described previously [4, 21, 29]. Prior to the experiment, dsDNA probes were prepared by annealing the 5′-fluorescein-labeled oligonucleotides to the unlabeled complementary oligonucleotides in a Biometra thermocycler (Analytik Jena, Jena, Germany). For the competition assay, a series of unlabeled oligonucleotide probes were synthesized and prepared. The dsDNA concentration was measured using a NanoDrop microvolume spectrophotometer (Denovix, DE, USA). To prepare the binding reaction mixtures, dsDNA probes were diluted to a final concentration of 3 µM and mixed with PIF3-bHLH proteins. The binding reactions were carried out in buffer D on ice for 30 min. Light exposure was avoided during incubation. Reaction mixtures along with EMSA loading buffer were then loaded into sample wells of a pre-run 10% native gel. The gel was then electrophoresed at 120 V for 80 min at 4 °C in 0.5 × TBE buffer. For visualization of the fluorescent band shifts, a FluorChem M system (ProteinSimple, MN, USA) was used to scan for fluorescence in the gel. Two repeats of fEMSA of wild type, H348A and R356A are presented in the Supplemental Fig. 2.

### Protein-DNA binding kinetic calculation

The protein–DNA binding kinetics parameters *K*_d_ and *B*_max_ were measured by quantifying fluorescent signals in well-defined band shifts. To obtain the values of *K*_d_ and *B*_max_, the intensities of the fluorescent-labeled DNA bands corresponding to protein–DNA complexes and free DNA probes were determined separately using ImageJ software. The following binding equation was used to calculate the protein–DNA complex fraction (also referred to as the bound fraction, BF%):$\:\text{BF}\% = \frac{\text{Bp}}{\text{UBp}}\:+\:\text{{Bp}} \times \text{100}\%$

The bound fraction is calculated by dividing the pixel value of DNA bands bound to the PIF3-bHLH protein (Bp) by the sum of the pixel value of all the DNA bands observed (represented by $\:\text{UBp}\text{\:+\:Bp}$, where UBp stands for unbound fraction). A nonlinear plot in which the BF% is plotted against the molarity of PIF3-bHLH was generated by fitting kinetic data with the equation given below:

$$\:\text{Y} = \frac{{\text{B}}_{\text{max}} \times \text{X}}{({\text{K}}_{\text{d}} + \text{X)}} $$

where X and Y represent the molarity of PIF3-bHLH and the corresponding BF%, respectively. The binding kinetics parameters *K*_D_ and *B*_max_ were estimated based on the equation above via nonlinear regression analysis using Prism software.

### Sequence alignment

To compare the bHLH region sequences of PIF3 and its homologs, we retrieved the PIF3 protein sequence from UniProt (UniProt accession number: 7D8T) and submitted it as an input query to NCBI’s BLAST search service. Sequence data available in the database were ranked according to their similarity to the query sequence. Homologous sequences with a score value between 95% and 99% were downloaded in FASTA format for comparative sequence analysis. Subsequently, the dataset, comprising the bHLH region sequences of PIF3 and its homologs in FASTA format, were subjected to multiple sequence alignment using the CLUSTALW server. Finally, the CLUSTALW output file was visualized and organized with BioEdit [38].

### Homology modeling and protein–DNA docking

To build a homology model of the AtPIF3-bHLH homodimer, we used the SWISS-MODEL server for template identification of the target sequence (UniProt ID: O80536), which comprised residues 340–397 of *Arabidopsis thaliana* PIF3 [47]. The search strategy was based on the sequence similarity between target and template. We conducted a survey of template coordinates available as Protein Data Bank (PDB) files. A template coordinate (PDB ID: 7D8T) was selected for homology modelling from a list of candidate models, considering the degree of sequence similarity and the model quality. In this case, the quaternary structure of the template was manually modified for oligomeric modeling of the target sequence. Based on the annotated template structure, a homology model of the dimeric PIF3-bHLH protein was built and assessed automatically in the SWISS-MODEL workspace. For protein–DNA docking, B-DNA coordinates were generated using the Web 3DNA 2.0 server [48]. Out of the 10 docked structures with top scores generated using the ZDOCK server, a final model bearing a strong resemblance to the solved structures of close homologs was used to obtain structural information [49]. The final results of protein–DNA docking were visualized using PyMOL (PyMOL molecular graphics system, Schrödinger, NY, USA).

### Isothermal titration calorimetry (ITC)

For label-free characterization of protein–DNA binding affinity, we performed ITC using MicroCal ITC200 (Malvern Panalytical, Malvern, UK). To prepare dsDNA for ITC measurements, complementary strands of G-box DNA were dissolved separately in ITC buffer (30 mM HEPES, 125 mM NaCl, pH 7.5) to a final concentration of 2 mM and mixed in equal volume ratios. The mixture was heated at 100 °C for 1 min and then cooled to room temperature for annealing. The concentration of dsDNA was determined with a NanoDrop microvolume spectrophotometer (Denovix, DE, USA). The final concentration of dsDNA was adjusted to 40 µM. For protein sample preparation, purified AtPIF3-bHLH was diluted with ITC buffer to a concentration of 400 µM. Each titration measurement comprised forty injections of 1.8–2.4 µl protein solution into sample cells containing G-box DNA with a stirring speed of 1000 rpm under constant cell temperature of 25 °C. The duration of each injection was 2 s with an interval of 180 s. Heat signals were recorded for each binding event with a reference power of 10 µCal/s and an initial delay of 60 s. The raw titration data derived from injection peaks were integrated to generate curves. From these curves, the thermodynamic parameters were calculated based on the one-binding-site model out using MicroCal Origin version 7.0 (Malvern Panalytical).

## Results

### Phylogeny reconstruction of PIFs in embryophytes

**Fig. 1:**
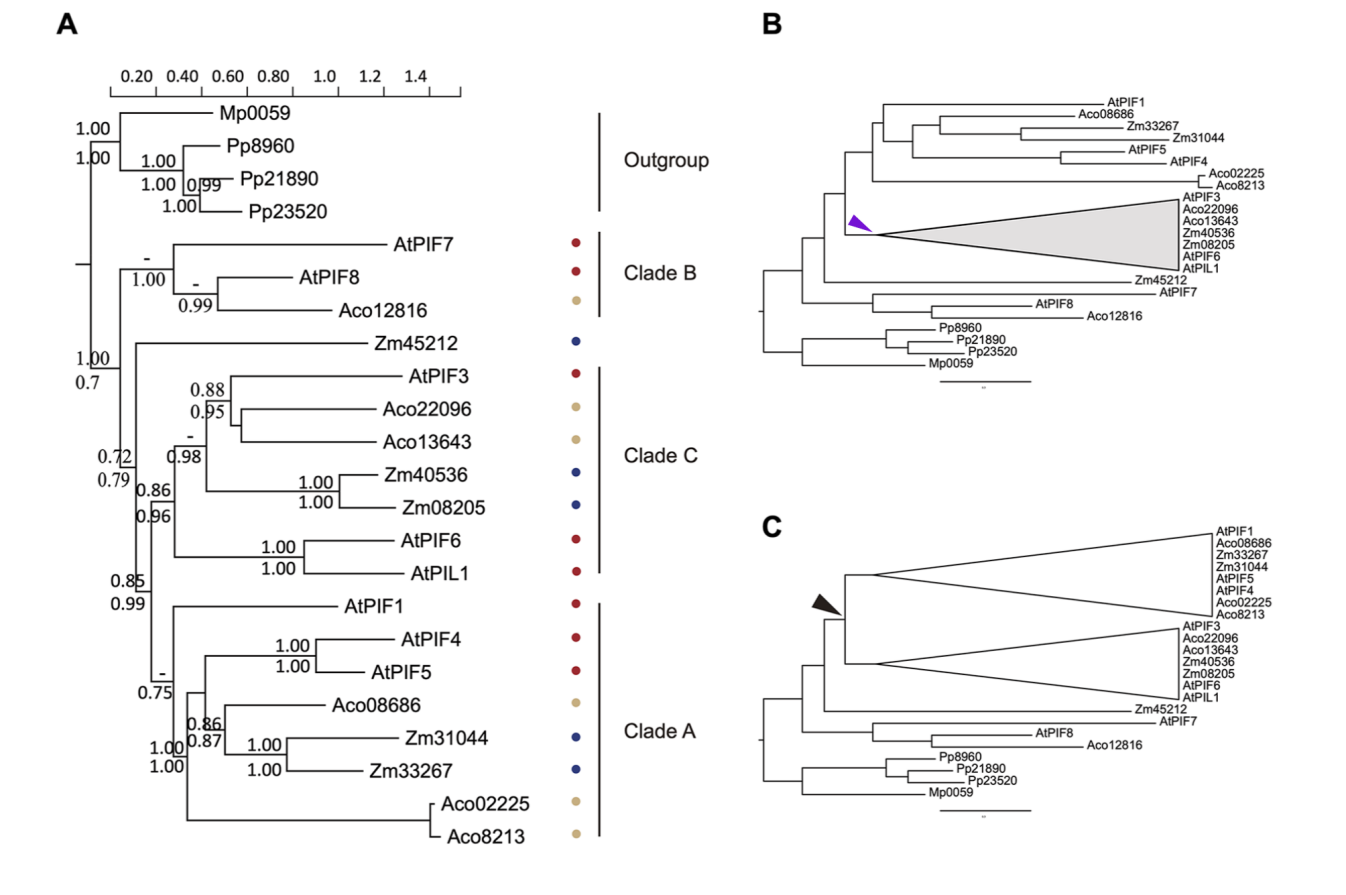
Phylogeny and evolutionary scenarios of embryophyte phytochrome-interacting factors (PIFs). (**A**) Clades A, B, and C are named following Jiang et al., 2022 [15]. The numbers above the branches denote approximate Bayesian inference values, and those below the branches denote approximate maximum likelihood ratio values. Green circles indicate PIFs obtained from *Arabidopsis thaliana*, light blue circles indicate PIFs obtained from *Z. mays*, and orange circles indicate PIFs obtained from *Ananas comosus*. (**B**) Evolutionary scenarios applied to test the selective pressure changes of the assigned clade (purple triangle). (**C**) Evolutionary scenarios applied to test diversification events of embryophyte PIFs. The black triangle indicates the diversification between clades A and C

To determine the relationships between PIFs identified from *Arabidopsis thaliana*, *Zea mays*, and *Ananas comosus*, PIFs obtained from *Marchantia polymorpha* and *Physcomitrium patens* were chosen as the root of the reconstructed PIF phylogeny (Fig. 1A). Three clades were identified: A, B, and C. Clade B diverged from outgroups first (Bayesian inference [BI]: 1.00; maximum likelihood [ML]: 0.7), following the divergence of Zm45212 (Bayesian inference [BI]: 0.72; maximum likelihood [ML]: 0.79) and then divergence between clades A and C (Bayesian inference [BI]: 0.85; maximum likelihood [ML]: 0.99). Clade B comprises AtPIF7, AtPIF8, and Aco12816 with branch support (Bayesian inference [BI]: –; maximum likelihood [ML]: 1.00). Clade A comprises AtPIF1, -4, -5, and orthologues that were identified from *Z. mays* and *Ananas comosus*, with moderate branch support (BI: 0.75; ML: –). In clade C, AtPIL1, AtPIF6, and AtPIF3, together with orthologues identified from *Z. mays* and *Ananas comosus*, are included with branch support (BI: 0.86; ML: 0.96). In this phylogeny, therefore, three monophyletic clades were identified with moderate to strong support. This topology was then subjected to CODEML to test the evolutionary scenarios of embryophyte PIFs.

### Patterns of molecular evolution among embryophyte PIFs

Two scenarios were used to infer the evolutionary pattern of embryophyte PIFs. The first was to test whether positive selection had acted on clade C; clade C was thus set as the foreground (Fig. 1B), while the remaining branches were regarded as background. The null model assumes all branches experienced the same selective pressure. The second scenario tested whether there were particular changes in pressure pre- and post-lineage diversification between clades C and A (Fig. 1C).

The estimated selective pressure for clade C was 0.32, which is slightly higher than that measured for the background branches (ω_b_ = 0.21; Table 1). When comparing pressure pre- and post-clade C and clade A diversification, the ω value changed from 0.19 (ω_pre−diversification_) to 0.28 (ω_post−diversification_). In addition, when taking the non-homogenizing selective pressure acting on amino acid residues into consideration, the ω value changed from 0.23 (ω_pre−diversification_) to 0.34 (ω_post−diversification_), with most amino acid residues subject to purifying selection (P_0_ = 0.19, P_2_ = 0.64; Table 1). Our results thus indicate that purifying selection acted among the clades of embryophyte PIFs.

**Table 1:** Evolutionary scenarios of embryophyte phytochrome- interacting factors (PIFs) with branch, branch–site, and site models

	np	DF.	likelihood	2ΔL (*p*)	ω
Branch model					
ω_C_ ≠ ω_b_	46	1	-38744.53	26.08 (*p* < 0.001)	ω_c_ = 0.32, ω_b_ = 0.21
ω_post−diversification≠_ω_pre−diversification=_ω_b_	46	1	-38745.24	24.66 (*p* < 0.001)	ω_post−diversification_ = 0.28, ω_pre−diversification_ = ω_b_ = 0.19
M0	45		-38757.57		
Clade model C					
ω_C_ ≠ ω_A_ ≠ ω_b_	50	2	-37966.29	3.7 (-)	ω_0_ = 0.29, P_0_ = 0.64; ω_1_ = 1, P_1_ = 0.17; ω_236_ = 0.06, ω_145_ = 0.05, ω_b_ = 0.06
ω_post−diversification≠_ω_pre−diversification=_ω_b_	49	1	-37955.81	24.65 (*p* < 0.001)	ω_0_ = 0.06, P_0_ = 0.19; ω_1_ = 1, P_1_ = 0.17; ω_post−diversification_ = 0.34, ω_pre−diversification_ = ω_b_ = 0.23, P_2_ = 0.64
M2a_rel	48		-37968.14		
Site model					
M2a	48	2	-38311.90	53.81	P_0_ = 0.46, P_1_ = 0.51, P_2_ = 0.02; ω_0_ = 0.21, ω_1_ = 1, ω_2_ = 9.2
M1a	46		-38338.80		

np: number of parameters, DF: degree of freedom, ω_C_, ω_A_, ω_b_: nonsynonymous to synonymous rates estimated from C clade, A clade and background. C clade and A clade corresponds to phylogenetic group inferred from Fig. 1.

### Nucleotide diversity changes among embryophytes and domain conservation of PIFs

**Fig. 2:**
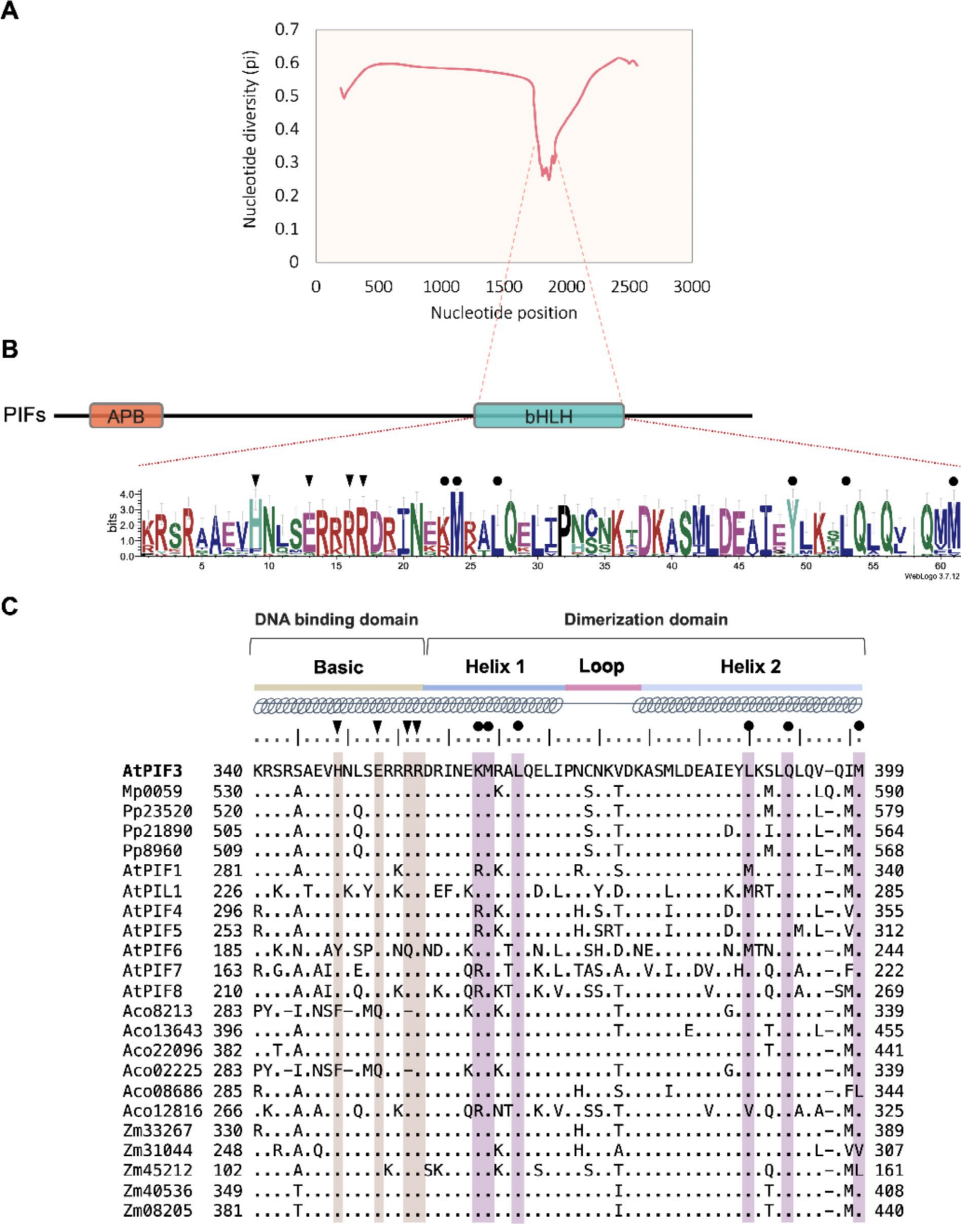
Changes in nucleotide diversity in the selected embryophyte phytochrome-interacting factor (PIF) sequences. (**A**) Nucleotide diversity curve measured from aligned PIFs. The diagram below the nucleotide diversity curve shows the PIF gene schema analyzed in this study. (**B**) Sequence logo conducted from aligned PIF basic helix-loop-helix (bHLH) regions. (**C**) Multiple sequence alignment of selected embryophyte bHLH regions. Triangles indicate four DNA-recognition sites in the basic region that have potentially been conserved across embryophytes. Circles highlight six potential conserved oligomeric state maintenance residues in the dimerization region. Numbers on the left side denote the first bHLH codon of the retrieved sequences. Numbers on the right side denote the end codon of the retrieved bHLH sequences

**Fig. 3:**
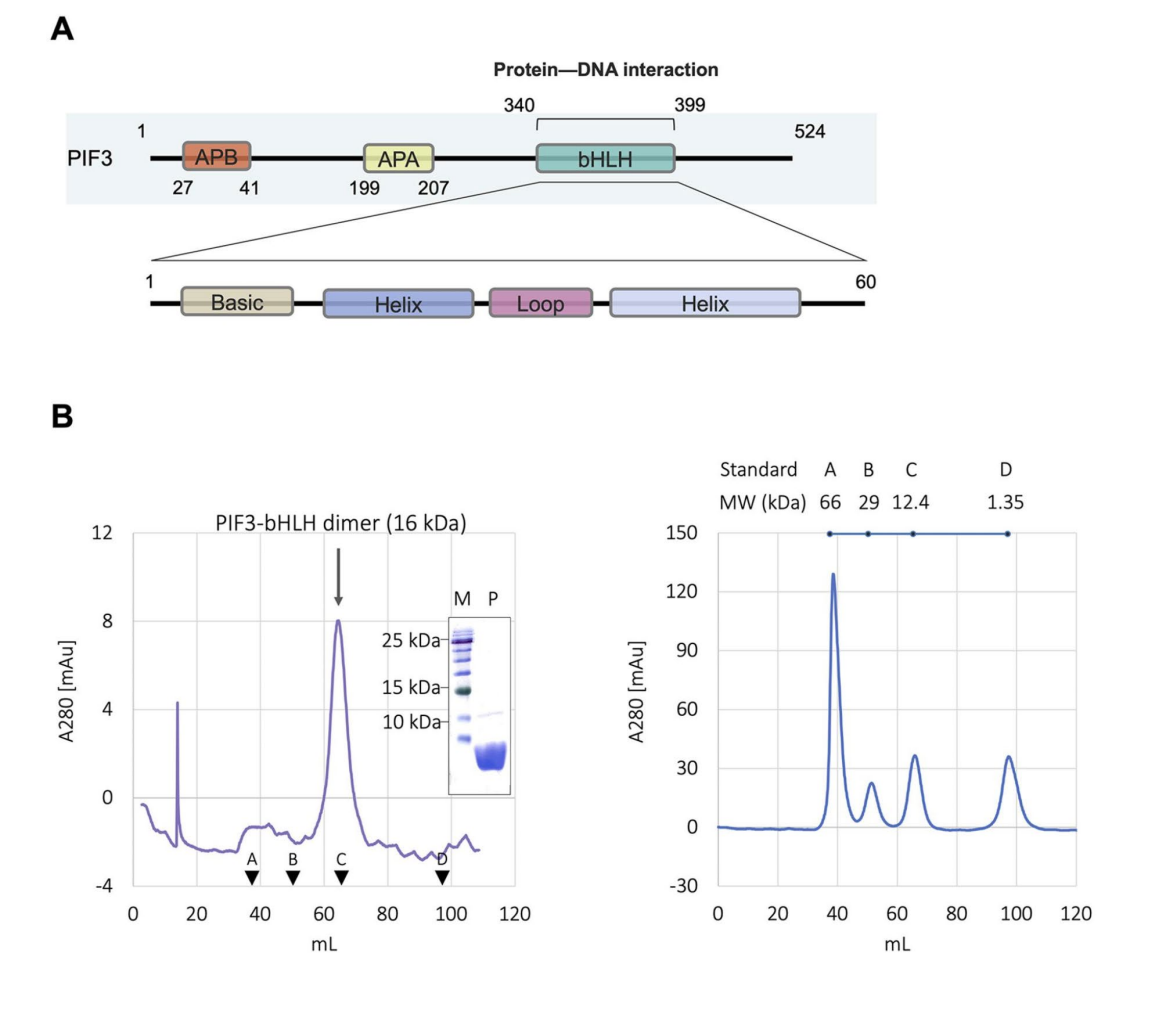
Purification and characterization of the PIF3-bHLH protein. (**A**) Schematic illustration showing the domain arrangement of the PIF3 protein. Functional domains are represented as boxes (box size is not proportional to domain length). The position of each functional motif and domain is indicated. (**B**) The elution profile of PIF3-bHLH from size exclusion chromatography (SEC). Elution peaks of molecular weight standards (cyan line) and PIF3-bHLH (black line) are marked. The elution peaks of molecular weight standards comprising albumin (66 kDa), carbonic anhydrase (29 kDa), cytochrome c (12.4 kDa), and vitamin B_12_ (1.35 kDa) are marked A, B, C, and D, respectively. On the right side of the SEC chromatogram, eluted PIF3-bHLH protein is visualized on SDS-PAGE gel via Coomassie blue staining (M and P represent marker and PIF3-bHLH protein, respectively)

**Fig. 4:**
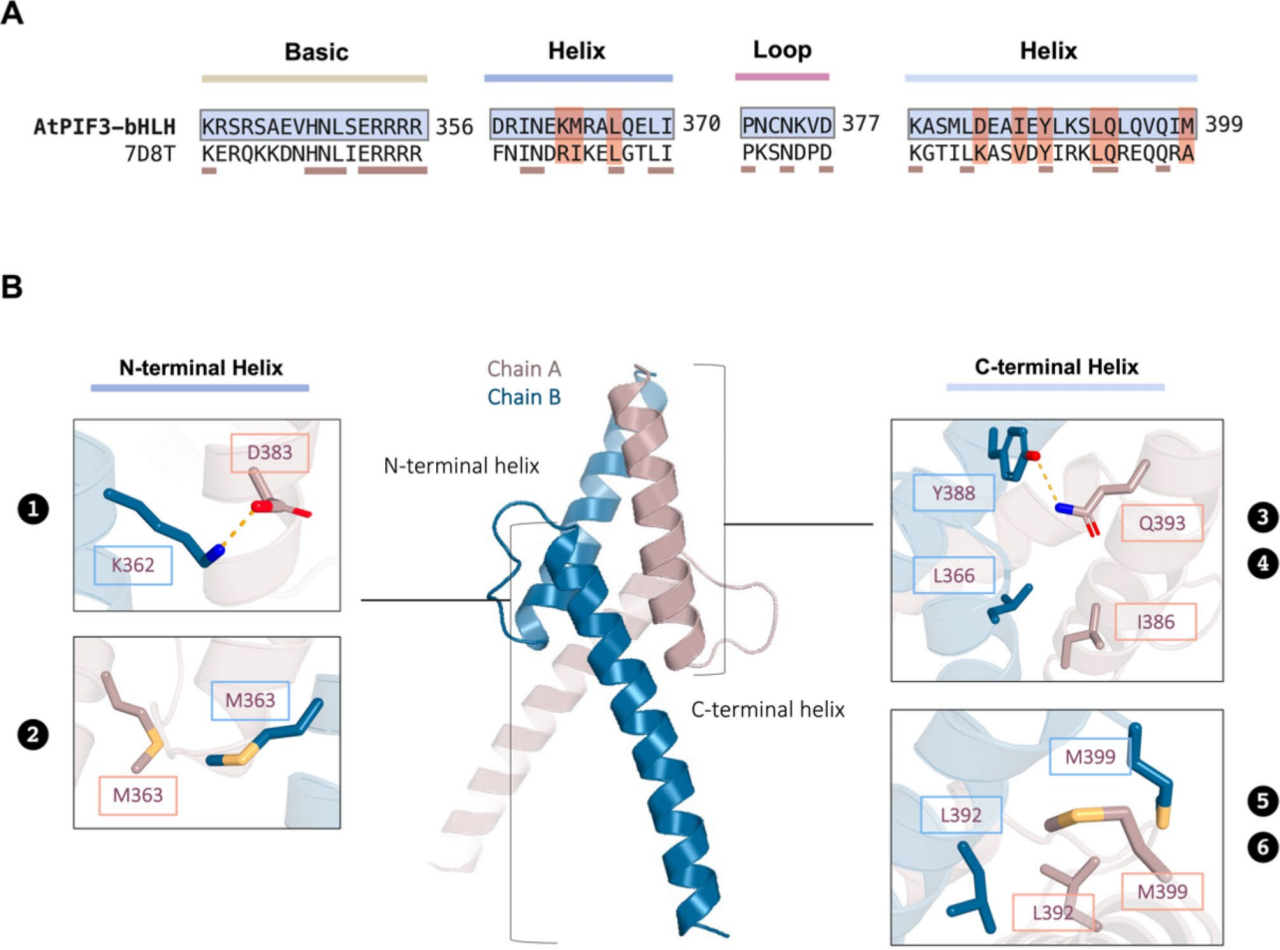
Structural details of the modeled dimeric structure of PIF3-bHLH. (**A**) Sequence alignment of PIF3-bHLH and template structure (PDB ID: 7D8T) with aligned residues underlined. Residues involved in dimer formation are highlighted in red. (**B**) The overall structure of the PIF3-bHLH dimer is displayed in the center with the N- and C-terminal helices indicated. A close-up view of residue contacts at the dimer interface is displayed on each side, with the number of interacting residue pairs marked in black circles

Our molecular evolution results show that embryophyte PIFs are under strong genetic constraints (Table 1). In the next step, a sliding window analysis was performed to reveal nucleotide diversity fluctuations along PIFs. The resulting nucleotide diversity curves showed a noticeable drop at 1785 bp, and this low nucleotide diversity continued to 1908 bp (Fig. 2A). Mapping the region of low nucleotide diversity back to the PIF protein sequence shows that it corresponds to the basic bHLH domain (Fig. 2A). The sequence logo of the bHLH domains extracted from the PIFs showed high amino acid residue conservation across embryophytes (Fig. 2B). Multiple sequence alignments were presented to further examine amino acid residue variations (Fig. 2C). In the DNA-binding domain, three sites (H348, E352, and R356) exhibited amino acid residue conservation across embryophytes (Fig. 2C, black triangles) and DNA-protein interaction potential in structural modeling (Fig. 3). The following amino acid residue conservation sites were also observed in the dimerization region: K362, M363, D383, I386, Y388, L392, Q393, and M399 (Fig. 2C, black circles). These sites are pivotal in maintain AtPIF3 homodimer in our structural modeling (Fig. 4). Because of this amino acid residue conservation, we selected the bHLH retrieved from *Arabidopsis thaliana* (AtPIF3) to use for examining bHLH oligomerization and bHLH–DNA binding preferences.

### Domain architecture and PIF3-bHLH protein dimerization

**Fig. 5:**
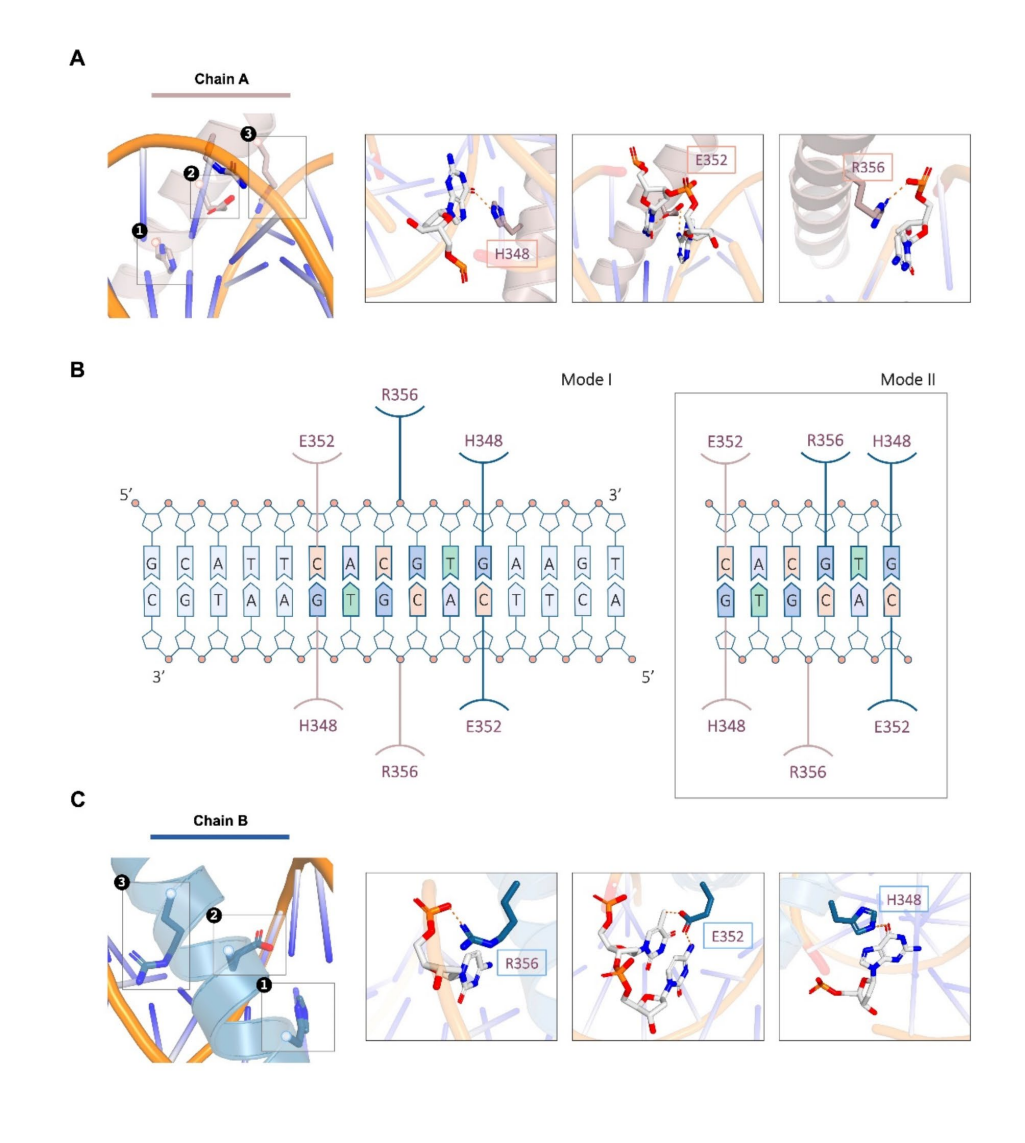
Structural details of AtPIF3-bHLH interacting with G-box DNA as revealed by protein–DNA docking analysis. (**A**) Close-up view of the AtPIF3-bHLH–G-box interaction interface of chain (**A**) Hydrogen bonds are indicated with dotted lines. (**B**) Summary of the interaction between AtPIF3-bHLH and G-box via modes I and II. (**C**) Close-up view of the AtPIF3-bHLH–G-box interaction interface of chain **B**. Hydrogen bonds are indicated with dotted lines

The PIF3 protein comprises 524 amino acids and is characterized by three functional domains: APB, active phytochrome A (APA), and bHLH (Fig. 5A). In this scheme, the bHLH domain responsible for protein–DNA interaction is located at the C-terminus of the PIF3 protein. Along with other PIFs harboring bHLH domains identified from *Arabidopsis thaliana*, *Z. mays*, and *Ananas comosus*, the 60-amino-acid bHLH domain in PIF3 features a basic region, which confers selectivity towards DNA target sequences, and a helix-loop-helix region, which stabilizes the dimeric structure.

To investigate the oligomeric forms of the PIF3-bHLH protein, size-exclusion chromatography (SEC) was performed using a Superdex75 size exclusion column (Cytiva, Marlborough, MA, USA). The UV trace at 280 nm showed a single major peak eluted at 64.46 ml (Fig. 5B). The sample was subjected to SDS-PAGE analysis to detect the presence of PIF3-bHLH in peak fractions. PIF3-bHLH was identified in the elution fraction corresponding to the peak at an elution volume of 64.8 ml. The molecular weight of the PIF3-bHLH eluted from the SEC analysis was 16 kDa. The fact that the PIF3-bHLH identified in the peak fraction and dimeric PIF3-bHLH (molecular weight of monomer = 7 kDa) are approximately identical in size confirmed that PIF3-bHLH forms a homodimer in aqueous solution. We then visualized the dimeric structure of PIF3-bHLH via homology modeling on the SWISS-MODEL web server. As exhibited by the 3D structure, which was simulated using a template structure with 35% sequence identity (Fig. 4A), each monomer comprises two amphipathic alpha helices connected by a loop with a length of seven residues (Fig. 4B). At the dimerization interface, putative intermolecular interactions are mainly mediated by residues D383, I386, Y388, L392, Q393, and M399 in the C-terminal helices of each monomer. In the proximity of the DNA-binding region, residues K362 and M363 in the respective N-terminal helices form contacts that stabilize the dimeric structure.

To confirm the role of the aforementioned amino acid residues in maintaining the intermolecular interactions between AtPIF3-bHLH monomers, a series of site-directed mutations including K362A, M363A, L366F, Y388A, L392H, and M399A was produced. The oligomerization states of each mutant were then analyzed via SEC. As shown in the elution profiles (See Supplemental Fig. 3A–F), the M363A, L366F, Y388A, and L392H mutants were eluted at the void volume, which indicates aggregate formation. In contrast, the K362A and M399A mutants exhibited a single elution peak corresponding to the dimer form. Together, these observations indicate that residues M363, L366, Y388, and L392 are crucial for the stability of the AtPIF3-bHLH homodimer, while K362 and M399 play a minor role in maintaining the dimeric organization.

### Docking simulation of protein–DNA interaction sites on the basic region forming contacts with G-box DNA

Homodimer formation is one of the key properties associated with bHLH regions. The dimeric structure is crucial for recognizing and interacting with its DNA targets, such as G-box. In the AtPIF3-bHLH homodimer, conserved DNA-interacting residues H348, E352, and R356 from each subunit mediate protein–DNA interaction, as shown in the docked structure of AtPIF3-bHLH binding to G-box DNA (Fig. 3). Although the two subunits confer different recognition specificity to each half of G-box (Fig. 3A), the basic region α-helices (residues 340–356) from each subunit take up position in the major groove of DNA in an identical manner (Fig. 3B). The docking results show that the H348, E352, and R356 side chains from one subunit are involved in base-specific interactions with the palindromic core G-box sequence (5′-CA*CG*TG-3′). The C and G nucleotides located at each end of G-box were recognized by chain A E352 and chain B H348, respectively, via either mode I or mode II (Fig. 3B). The difference between mode I and mode II was in the core region-binding mode. In mode I, the R356 located on chain A and R356 on chain B made contact with the phosphate backbone. In mode II, the R356 on chain A made contact with the phosphate backbone, while the R356 on chain B made contact with the G base.

### Site-directed mutation of protein–DNA interaction sites on basic region exhibited minor influence on protein–DNA binding ability

**Fig. 6:**
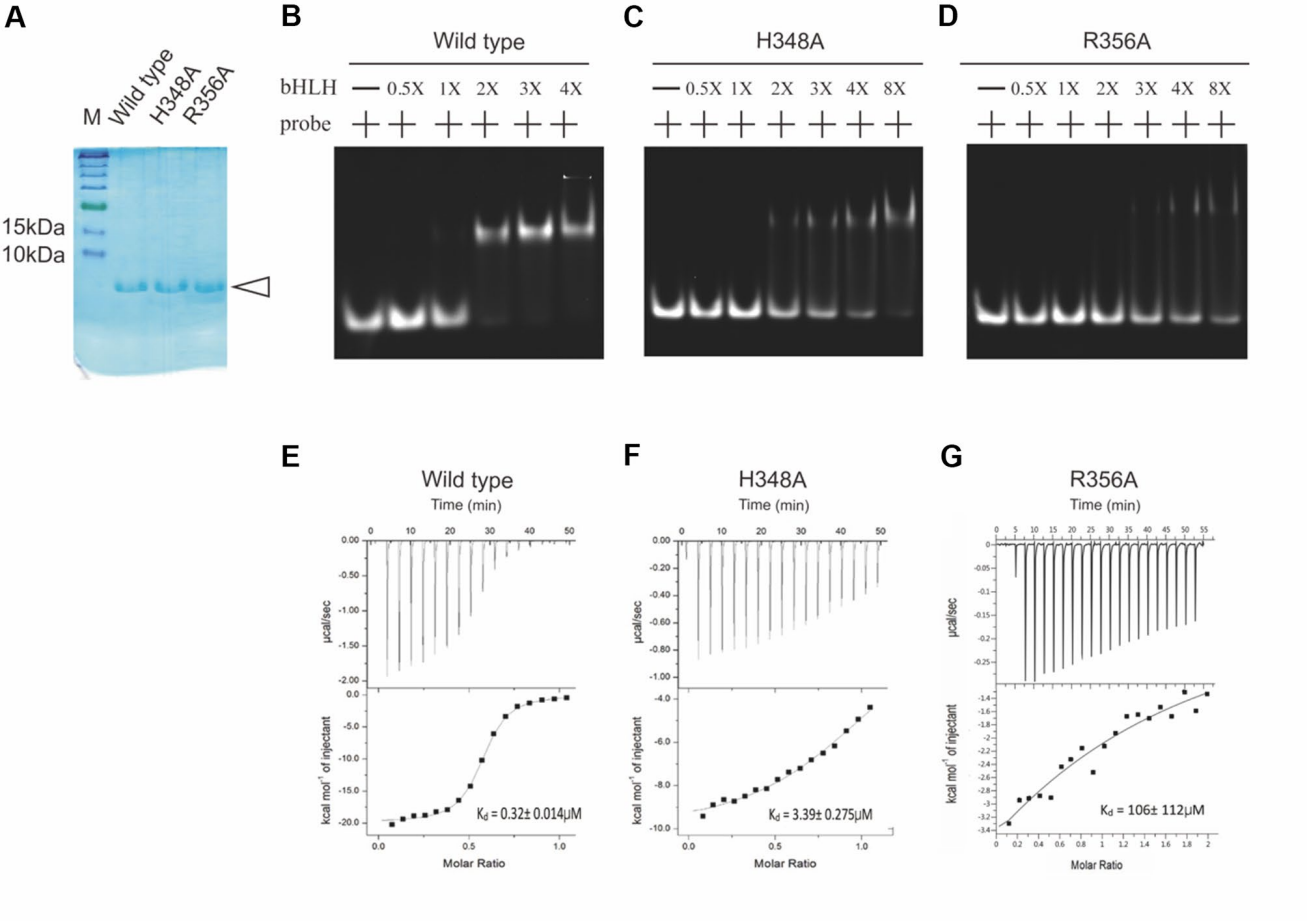
Comparative analyses of the DNA-binding ability of AtPIF3-bHLH wild-type (WT) and mutants using ITC technique. (**A**) Purified WT, H348A and R356A recombinant protein were each adjusted to 1.2 µg/µl. (**B – D**) Band-shift profiles of (**B**) WT, (**C**) H348A and (**D**) R356A mutants binding to fluorescein-tagged G-box probe at the concentrations indicated. Original gels of A-B correspond to Supplemental Figure S2. (**E–G**) ITC profiles of (**E**) WT, (**F**) H348A, and (**G**) R356A, including binding constants (*K*_d_) calculated from the fitted curves

From the docking results, we surmised that the DNA-interacting residues H348 and R356 are responsible for base recognition in the flanking and core regions of G-box DNA, respectively. To assess the influence of H348 and R356 on the DNA-binding activity of AtPIF3-bHLH towards G-box, we conducted site-directed mutagenesis to produce AtPIF3-bHLH mutants H348A and R356A. We then conducted preliminary experiments to characterize the DNA-binding activity of H348A and R356A towards G-box DNA (5′-GCATT*CACGTG*AAGT-3′). Wild-type AtPIF3-bHLH (WT) was used as a positive control. The binding patterns of WT, H348A, and R356A were revealed using fEMSA (Fig. 6A and B). Analysis of WT interacting with the G-box sequence revealed that the bound fraction increased with an increase in the molar ratio of DNA to protein from 1:1 to 1:20. For H348A and R356A, impaired binding to the G-box sequence was indicated by the presence of unbound fractions at a 1:10 molar ratio of DNA to protein.

We used isothermal titration calorimetry (ITC) to further investigate the binding affinity of WT, H348A, and R356A to G-box DNA (5′-AGGAA*CACGTG*ACCC-3′). In the ITC measurements, we recorded heat generated during serial titrations of DNA into sample cells containing purified protein (Fig. 6C–E). With each titration experiment, we observed a slight decrease in heat signal, indicating saturation of the available binding sites. Of the three interaction pairs, the binding curve for the H348A–G-box interaction was unsaturated. For curve fitting, the injection peaks of each thermogram were integrated and applied to a single-binding-site model. The shape of the ITC curve varies depending on whether WT, H348A, or R356A is interacting with the G-box DNA, suggesting differential binding affinity. Based on nonlinear regression analysis, the dissociation constant (*K*_d_) obtained for WT interacting with G-box was 0.32 ± 0.014 µM. For H348A–G-box and R356A–G-box, however, *K*_d_ was 3.39 ± 0.275 µM and 106 ± 112 µM, respectively. The fact that *K*_d_ for the mutants was almost 1000 times that of WT suggests partial binding ability.

### Implication of transcriptional regulatory divergence from the binding preferences of PIF3-bHLH towards E-box variants

Given the wide distribution of E-box (5′-CANNTG-3′) in the promoters of PIF3-bound genes [4], the recognition of E-box variants is an intimate part of PIF3-mediated transcriptional regulation. Among the PIF3-bound DNA motifs, G-box (5′-CACGTG-3′) and PBE-box (5′-CACATG-3′) were predominant. We also identified other E-box variants and tandem repeats of E-box (also termed tandem E1-E2 consensus elements [50]) in the promoters of PIF3-bound genes.

**Fig. 7:**
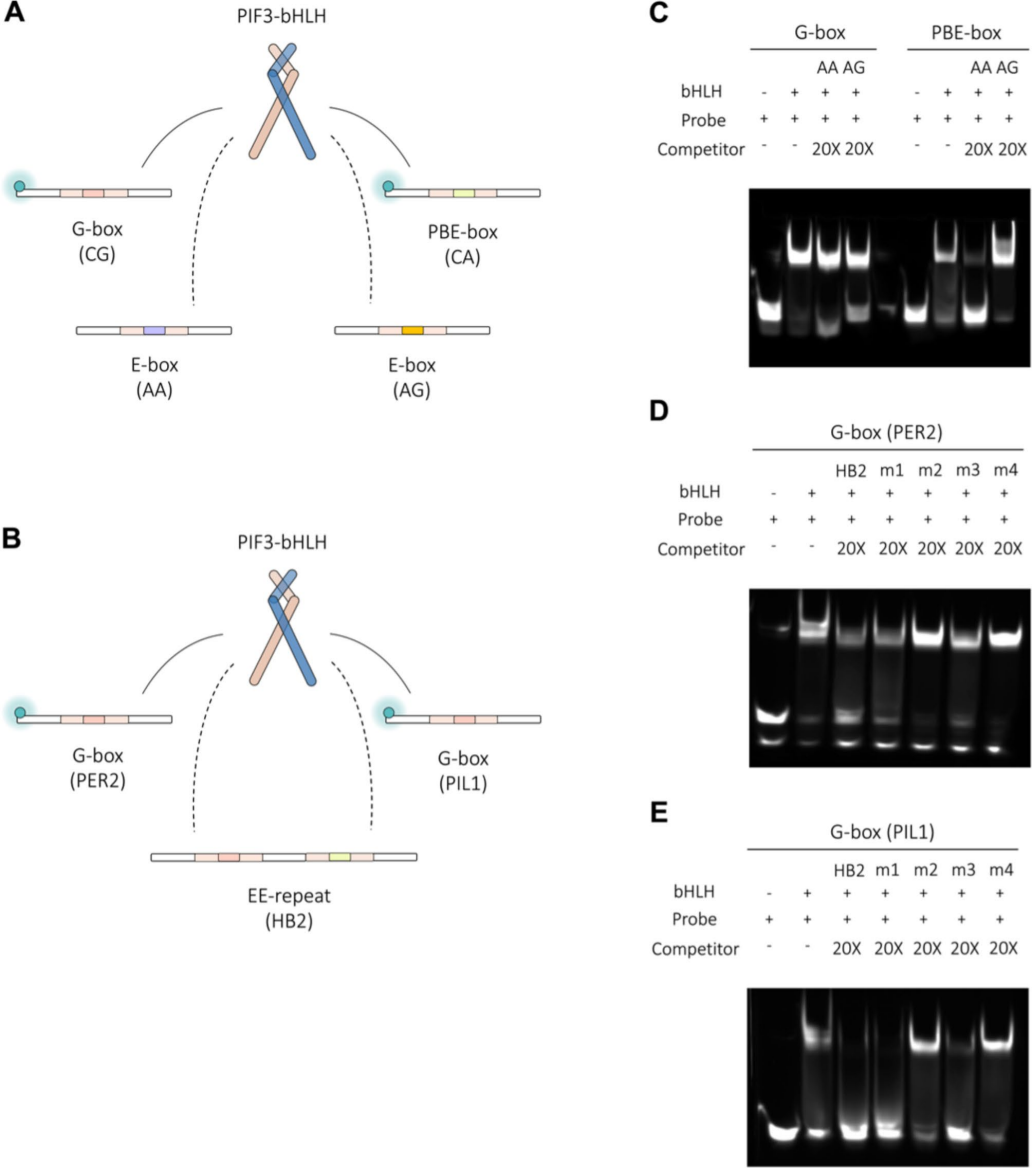
Determination of binding preference of PIF3-bHLH toward E-box variants via fluorescence-based electrophoretic mobility shift assay. (**A**–**B**) Schematic diagram of binding interaction between AtPIF3-bHLH and its target DNA motifs: G-box, PBE-box, E-box, and E1-E2 elements. (**C–E**) Fluorescence imaging of EMSA gels revealing the band-shift pattern of AtPIF3-bHLH binding to 5′-fluorescein-labeled probes. Original gels of C-E correspond to Supplemental Figs. 13–15. A 20-fold excess of competitor was added in each binding reaction

How is specific recognition of similar DNA motifs achieved, given the redundant use of DNA motifs in functionally diverse genes? To address this question, we characterized the binding preference of AtPIF3-bHLH in vitro using E-box variants found in the promoters of three well-characterized genes (*PIL1*, *HB2*, and *PER2*) as DNA probes (Supplemental Table 1). *PIL1* and *HB2* are direct target genes of PIF3, whereas *PER2* serves as a target for CLOCK-BMAL1 in the mammalian circadian clock. We thus designed two sets of competitive fEMSA experiments (Fig. 7A and B). In the first, we analyzed the binding of AtPIF3-bHLH to a 5′-fluorescein-labeled G-box and PBE-box in the presence of other E-box variants (we used two randomly selected variants, CAAATG and CAAGTG, referred to as AA and AG, respectively, as competitors).

The second experiment involved first characterizing the binding activity of AtPIF3-bHLH toward 5′-fluorescein-labeled G-box in the presence of tandem E1-E2 elements and a series of mutants and then assessing the influence of flanking regions adjacent to G-box on binding preference. AtPIF3-bHLH preferentially binds to G-box DNA in the presence of either E-box variant as a competitor (Fig. 7C). Intriguingly, we observed different band shift patterns when we substituted G-box DNA with PBE-box DNA. In this scenario, a DNA band indicating free probes containing PBE-box was present when AA was the competitor, but this band shifted in the opposite direction when AA was replaced with AG. This indicates that combinations of G-box, PBE-box, and E-box may influence the selectivity of AtPIF3-bHLH. Investigating the binding preference of AtPIF3-bHLH for G-box (*PER2*) when tandem E1-E2 elements were present as competitors revealed the predominant interaction of AtPIF3-bHLH with G-box (Fig. 7D). In contrast, an alteration in band-shift pattern was observed when G-box (*PER2*) was substituted with G-box (*PIL1*), suggesting that the flanking sequence also plays a role in determining binding preference (Fig. 7E). The nucleotide composition of bHLH binding motifs is therefore the key factor in establishing bHLH–DNA recognition priority.

## Discussion

### Embryophyte sequence conservation and post-divergence relaxation from genetic constraint

The PIFs are regulatory hubs for precise molecular networks that integrate external and internal signals in land plants [3, 15, 19]. For example, AtPIF3 is known to interact with *ARF18*, *AtPIF2*, *CBF* (C-repeat binding factors), *HDA15*, *PIL1* (phytochrome interacting factor 3-like 1), *phyB*, and *TOC1*, to govern the growth of *Arabidopsis* [3, 4, 18, 21]. Maintaining sequence-recognition features is key when interacting with such diverse genes. In other words, a key gene like *AtPIF3* is assumed to be subject to purifying selection. The results of our evolutionary analysis have provided supporting evidence for this (Table 1; branch model, ω_c_ = 0.32). Further, the almost-zero ω values that we obtained for clades A and C under the clade model C scenario (Table 1; ω_C_ = 0.06, ω_A_ = 0.05) and the somewhat higher ω values estimated post-diversification than pre-diversification suggests a relaxation from genetic constraint following rapid fixation of the clade A and clade C PIFs. In the Plantaginaceae, both *CYCLOIDEA* (*CYC*) and its paralogue, *DICHOTOMA* (*DICH*), are maintained, and both contribute to establishing floral symmetry, because of relaxed purifying selection [51]. Based on these findings and our evolutionary analysis of PIFs, we postulate that functional genes may obtain their diverse functions during the duplication window and then rapidly fix on the beneficial functions that ensure the survival of the species.

After the divergence, the PIFs and their homologues exhibit expression level differences and functional divergence either in light response or in seedling growth stages [52–54]. In *Arabidopsis*, PIF1 mRNA is more abundantly expressed than other PIF mRNAs in imbibed seeds, reflecting its key role in seed germination. During the early stages of seedling development, PIF1 and PIF3 mRNAs show higher expression levels compared to PIF4 and PIF5 mRNAs, but their expression declines as the seedlings mature. Moreover, PIF1 and PIF3 are regulated through light-induced degradation, which influences the photomorphogenesis of *Arabidopsis* seedlings [55, 56], while PIF4 and PIF5 play crucial roles as both photoreceptors and thermosensors [57]. PIF homologues identified in *Solanum lycopersicum* include 8 PILs (PIF-LIKEs) with an APB motif, with 3 of these PILs also containing an additional APA motif [54]. Purifying selection signals are detected of most SlPIFs, except the SlPIF7b. A significant positive selection signal is detected, hinting the functional divergence. Among these identified *S. lycopersicum* PILs, the response to light exposure exhibit gene expression pattern variations. For example, light stimulated the expression of SlPIF1a, SlPIF4, SlPIF7a and SlPIF7b, while the expression levels of SlPIF1b and SlPIF3 were significantly decreased in response to light exposure [54]. In summary, PIFs and their homologues exhibit varying rates of nucleotide substitution, which contributes to genetic diversity during duplication events. Meanwhile, advantageous functions such as those related to photomorphogenesis and thermomorphogenesis are rapidly preserved through purifying selection.

We identified a region of low nucleotide diversity among the selected PIFs (Fig. 2A). This region corresponds to the bHLH domain, which is known for binding directly to G-box or E-Box [3, 23, 25, 31]. This suggests that the bHLH region was subject to stronger purifying selection than other PIF regions. The reason may be related to the bHLH binding mechanism, which involves forming a mirror image-like protein complex to bind its target nucleotides. To bind to its target nucleotides in G-box or PBE-box, bHLH forms either a homodimer or heterodimer [31, 58]. For example, the bHLH domain of E47 and NeuroD1 form a heterodimer to bind PBE-box (5′-CATCTG-3′) [26]. The loop region of E46 and NeuroD1 differs, but there are similarities in their basic regions and the other two helix regions in humans [26]. In *Arabidopsis thaliana*, the bHLH region of AtPIF4 bind its target, G-box (5′-CACGTG-3′) in either homodimer or homotetramer form [17, 59]. Both cases suggest that maintenance of a dimer is key prior to DNA recognition. The conserved amino acid residues identified in the DNA-binding domain (H348, E352, R355, and R356) and the dimerization region (K362, M363, L366, Y388, L392 and M399) across embryophytes and structural modeling not only hint at the ancient origin of the dimer formation of plant bHLH regions, but also suggest ancient establishment of the bHLH–DNA binding mechanism (Figs. 2C, 4 and 3). However, the bHLH exhibit a broad range of possibility in forming polymer and DNA recognition. For example, the AtPIF4 bHLH forms homotetramer via E275, R276, K278, Q281, E282, Q311 and W314. In addition, the YUCCA8 G-box is recognized by the AtPIF4 basic region in an asymmetric form [59]. The mechanism of bHLH polymerization and DNA recognition preference can be revealed by examining the relevant amino acid resides, and the results can be generalized across embryophytes due to conservation at the amino acid residue level.

### Sequence conservation of bHLH domain mediating self-oligomerization through dimerization

The bHLH domain has been reported to form either homodimers or heterodimers via the Helix 1 and Helix 2 regions in animals and plants [24, 25, 31]. Here, we modeled AtPIF3-bHLH to explore the mechanism behind the dimerization of bHLH in plants. The modeled homodimer showed that the monomers contact one another via key residues (K362, M363, L366, Y388, L392, and M399) in the dimerization region (Fig. 4B), forming a helical bundle structure. It has been proposed that this helical bundle structure contributes to its stability, and the structure formed by MYC2-bHLH further supports this idea [24].

The site-directed mutagenesis showed that four of the amino acid residues are critical for maintaining dimer form (Supplement Fig. 1). This may be related to historical residue conservation among embryophytes. Four key residues (M363, L366, Y388, and L392) are almost identical across embryophytes, suggesting the inevitable roles of these amino acid residues in dimer formation (Fig. 2C, purple circles). Considering the purifying selection of PIFs and low nucleotide diversity in the bHLH region we observed, we postulate that these amino acid residues are so crucial for maintaining the structure of the helical bundle that any substitution thereof among embryophytes is unlikely.

### The reduced binding affinity values and conserved DNA-recognition sites across embryophytes suggests evolution of best-fitting DNA-recognition sites

We attempted to express the four known DNA-recognition sites (H348, E352, R355, and R356) as mutant recombinant proteins via our expression process. However, only two of the four were synthesized successfully (H348A and R356A). The other two failed, which may also hint at the invariant nature of these two sites. The binding ability and affinity of both H348A and R356A were lower than those of WT (Fig. 6), suggesting that these sites are critical for target DNA recognition. MYC2-bHLH–G-box also exhibits the DNA-recognition ability of these amino acid residues, but the importance of these residues across embryophytes has been less discussed [24]. Examining the alignment of both PIF and MYC-bHLH nucleotides revealed that the same four amino acid residues (H348, E352, R355, and R356 in AtPIF3) exhibited almost identical patterns both across embryophytes and between gene groups (Supplemental Fig. 4). Integrating our binding-ability and sequence-alignment data, we postulate that these four amino acid residues evolved prior to the emergence of *M. polymorpha* and then rapidly became fixed in embryophytes due to their specific DNA-recognition ability.

### Core region recognition ability contributes to AtPIF3-bHLH–G-box interaction

The modeled AtPIF3-bHLH–G-box protein–DNA interaction model exhibited a symmetrical binding mode (Fig. 3). The first significant feature revealed by our modeling was the specific recognition of the C and G bases at the ends of G-box (5′-*C*ACGT*G*-3′) via H348 and E352 of each chain, in both mode I and mode II (Fig. 3). The second was the dynamic recognition of G-box core regions by AtPIF3-bHLH. In mode I, both the arginines located on chain A (R356) and chain B (R356) made contact with the phosphate backbone. In mode II, however, the arginine located on chain B (R356) formed hydrogen bonds with a guanine base, and the arginine on chain A (R356) made contact with the phosphate backbone. Compared with our findings from other known bHLH–G-box protein–DNA complexes, such as MYC2–G-box, MITF–G-box, and PHO4–G-box, the dynamic binding mode found here is unique [24, 30, 60], particularly compared to core region binding preferences mentioned in both plant and animal studies [23, 29–31, 60]. For example, PHO4–bHLH binds to the central G of the core region (5′-CAC*G*TG-3′) via hydrogen bonds. An exception is the MITF–DNA recognition structure, which exhibits no specific binding to the core region; it simply recognizes the C and G bases and forms hydrogen bonds to interact [60]. Based on our modeled bHLH–G-box structure and previous studies, we assume that the C and G bases at the ends of G-box (5′-*C*ACGT*G*-3′) play a key role in bHLH-DNA recognition coupled with flexible core region recognition. The dynamic core region recognition ability of AtPIF3 bHLH may be the mechanism behind the differences in the binding ability of AtPIF3-bHLH–E-box (5′-CANNTG-3′).

### Recognition ability of diverse DNA motifs may allow AtPIF3 to serve as a molecular regulatory hub in circadian rhythm

AtPIF3 is known to be involved in not only light signaling and circadian rhythms, but also in cold acclimatization [19, 20, 61]. Multiple genes have been found to interact with AtPIF3, such as *TOC1*,* PIL1*,* HFY1*, and *ARF18* [3, 4, 18, 19]. PIFs have therefore been studied to unveil their roles in light-induced regulatory networks [3, 19]. Using Chip-seq and micro-array techniques, the G-box (5′-CACGTG-3′), PBE-box (5′-CANNTG-3′), and tandem E1-E2 repeats were confirmed as bHLH binding targets, but the bHLH–DNA recognition mechanism was less discussed. Our results show that the nucleotide composition of the core region, the flanking regions, and E-box repeats is a key factor in determining bHLH–DNA binding ability (Fig. 7). Among the examined DNA motifs, a core region containing CG is the dominant motif, suggesting the G-box (5′-CACGTG-3′) binding preference of AtPIF3–bHLH, which prefers CG, AA, CA, and AG, in descending order. However, we also observed an exception: the tandem E1-E2 repeat can also outcompete the G-box (Fig. 7E: HB2, m1, and m3 lanes), suggesting flexible recognition abilities of the bHLH DNA-binding motif. This phenomenon may be related to the core region recognition flexibility of AtPIF3-bHLH (Fig. 3).

This core region recognition flexibility of AtPIF3-bHLH and the results of our competitive assays may hint at an evolutionary preference for G-box. If this is the case, G-box should be dominant in the genome; we thus selected 53 circadian rhythm-related genes^4^ and screened their corresponding upstream nucleotides for G-box (Supplemental Figs. 5–12). Genes containing G-box outnumbered those that did not (36 vs. 17; Supplemental Fig. 6), which supports our hypothesis that G-box is favored.

Another significant feature is the amino acid residue conservation across embryophytes (Fig. 2), which suggests functional constraints among plant bHLH domains. Once the beneficial trait of asymmetric binding was established, it was quickly fixed across embryophytes. As a consequence, the PIFs became involved in a diverse regulatory network. Considering their sequence conservation across embryophytes and bHLH–DNA binding preference, we propose that bHLH–DNA recognition flexibility related to diurnal rhythm regulation may have evolved across embryophytes.

## Conclusions

AtPIF3 is a crucial mediator in photomorphogenesis and other molecular pathways, such as the circadian clock. Recognizing diverse types of target DNA motifs allows it to participate in diverse molecular pathways and regulate its direct target genes downstream. Through molecular analysis, point mutations in the bHLH domain, and competitive fEMSAs, we have identified the binding preferences of AtPIF3-bHLH. Further, the conserved protein–protein interaction sites of bHLH across plants suggest an ancient origin for bHLH dimerization.

## Electronic supplementary material

Below is the link to the electronic supplementary material.

Supplementary Material 1

## Data Availability

Not applicable.
