# Supplementary material for: Specific binding between Arabidopsis thaliana phytochrome-interacting factor 3 (AtPIF3) bHLH and G-box originated prior to embryophyte emergence
Source: BMC Plant Biol. 2024 Nov 11;24:1060. doi: 10.1186/s12870-024-05777-z (PMC11552376; doi:10.1186/s12870-024-05777-z)
Supplement: Supplementary file 1 — Supplementary Material 1 [file 12870_2024_5777_MOESM1_ESM.docx]

Supplemental Information

**Supplemental Table 1**. DNA probes used in fluorescence-based electrophoretic mobility shift assay (fEMSA)

|  | Probe | Sequence | Label |
| --- | --- | --- | --- |
| Experiment I | pPER2 (G-box) | AGGAACACGTGACCC | 5′ fluorescein |
|  | CA (PBE-box) | AGGAACACATGACCC | 5′ fluorescein |
|  | AA (E-box) | AGGAACAAATGACCC | Unlabeled |
|  | AG (E-box) | AGGAACAAGTGACCC | Unlabeled |
| Experiment II | pPER2 (G-box) | AGGAACACGTGACCC | 5′ fluorescein |
|  | pPIL1 (G-box) | GCATTCACGTGAAGT | 5′ fluorescein |
|  | pHB2 | GATCCACGTGAAGTTCACATGCTTT | Unlabeled |
|  | m1 | GATCCACGTGCCGTTCACATGCTTT | Unlabeled |
|  | m2 | GATCCAAATGAAGTTCACATGCTTT | Unlabeled |
|  | m3 | GATCCACGTGAAGTTCAAATGCTTT | Unlabeled |
|  | m4 | GATCCAAATGAAGTTCAAATGCTTT | Unlabeled |


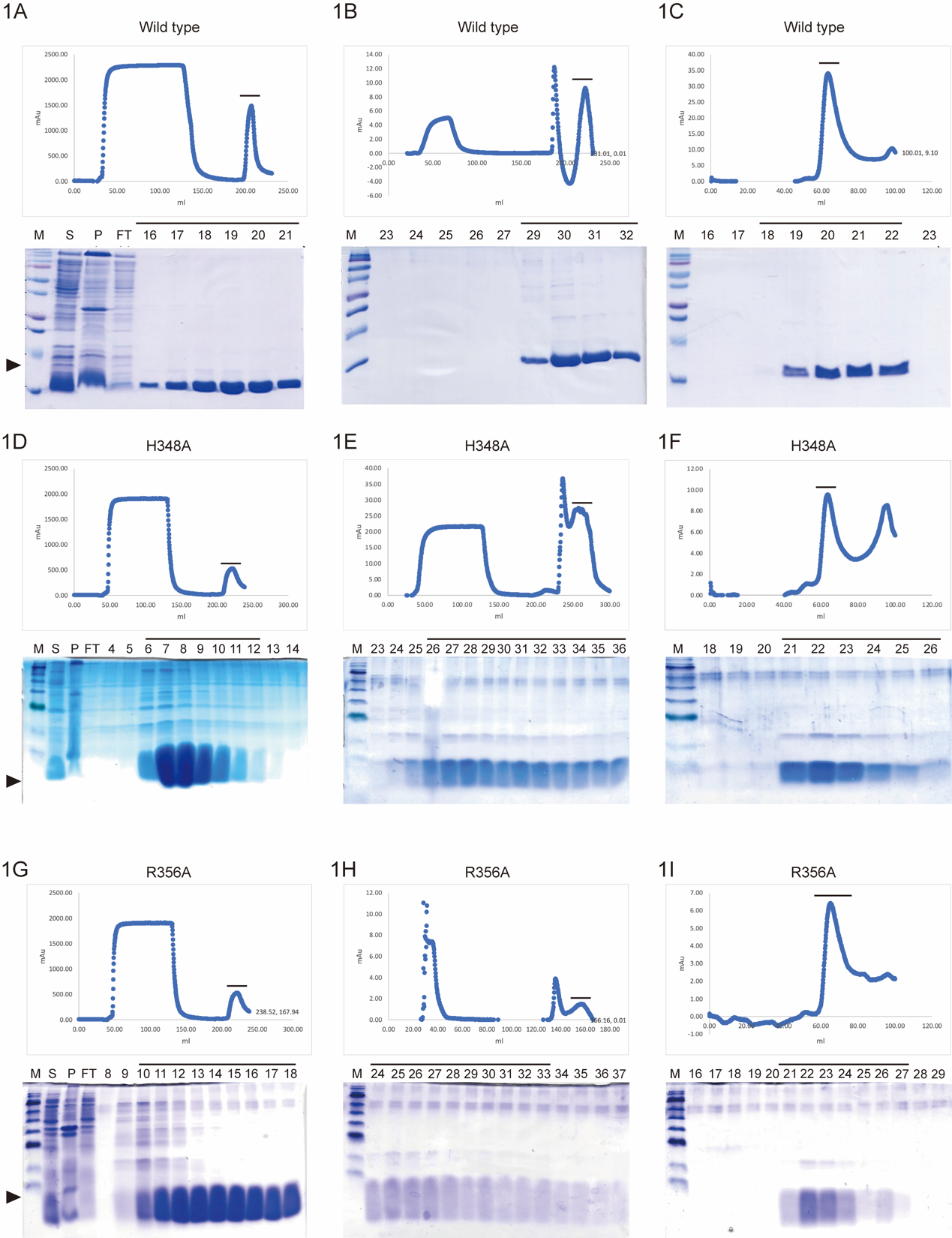


**Supplemental Figure 1.** Purification profiles and SDS-PAGE gels obtained from wildtype, H348A and R356A recombinant protein purification process.

M: marker. S: supernatant. P: pellet. FT: flow through. Numbers above each lane: the fraction collected. Black solid line: the elution range collected for recombinant protein concentration.


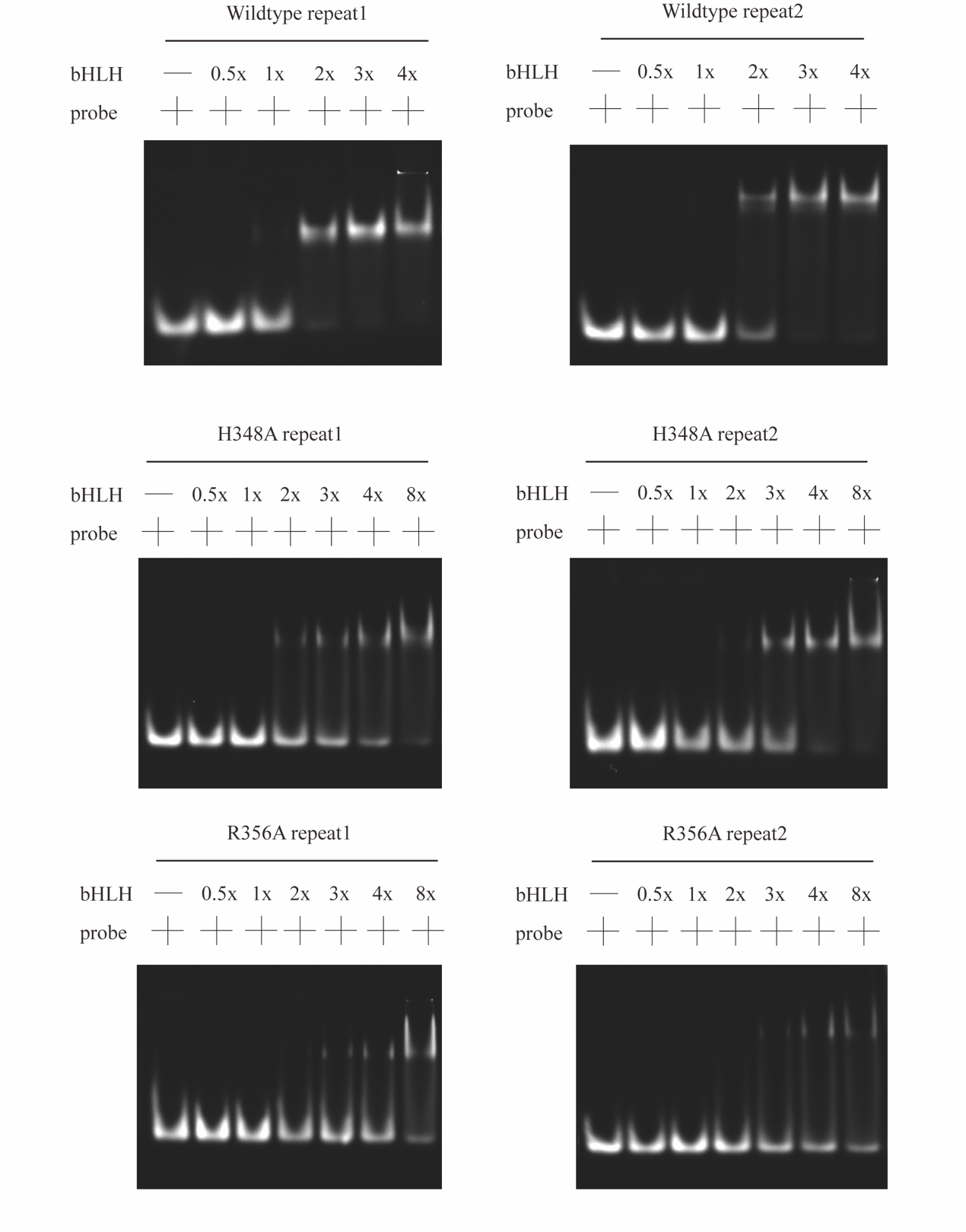


**Supplemental Figure 2.** Two repeats of wildtype PIF3 bHLH, H348A and R356A recombinant proteins fEMSA assays.


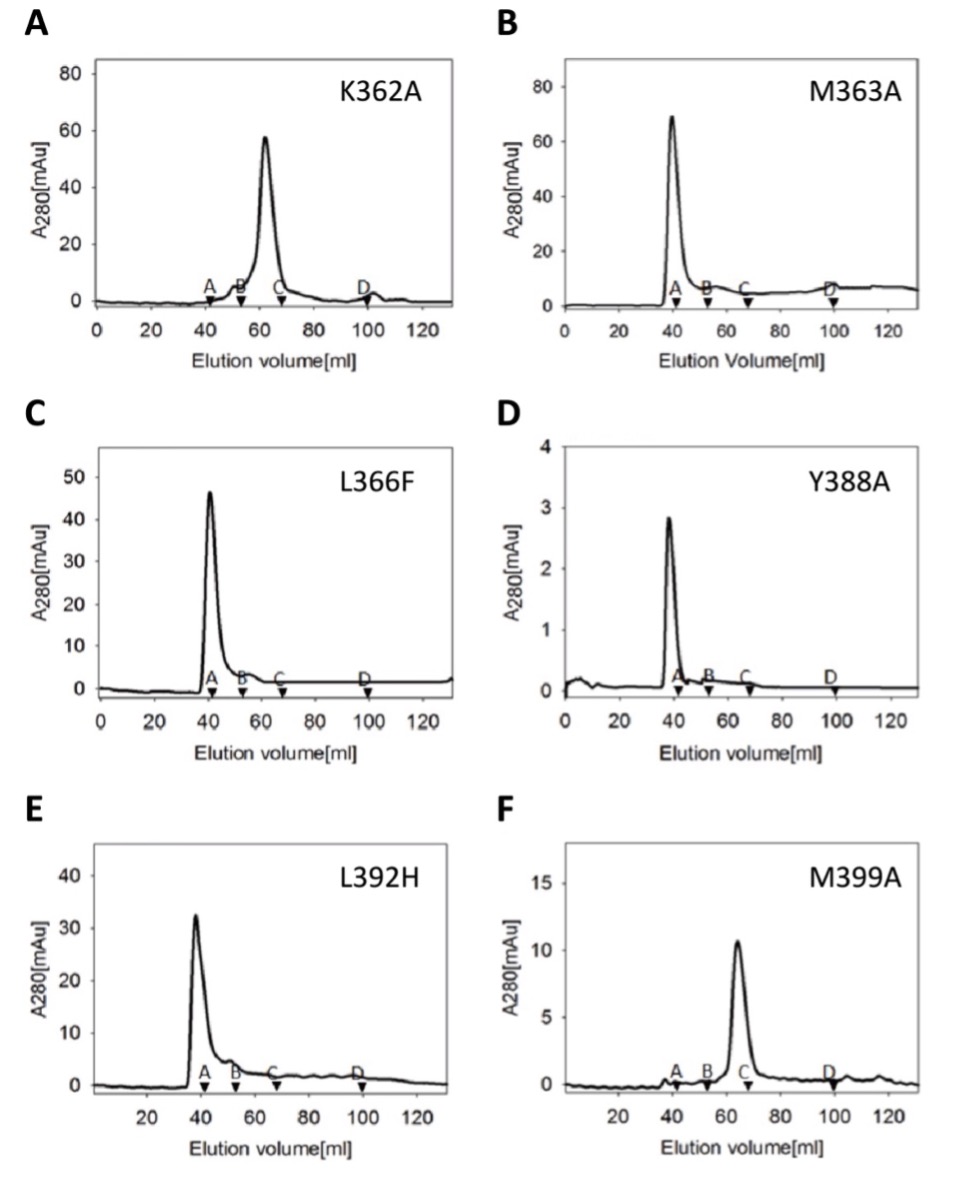


**Supplemental Figure 3**. Determination of oligomeric states of AtPIF3-bHLH mutants via size-exclusion chromatography (SEC).

(**A–F**) SEC elution profiles of AtPIF3-bHLH mutants: (**A**) K362A; (**B**) M363A; (**C**) L366F; (**D**) Y388A; (**E**) L392H; (**F**) M399A. Elution volumes corresponding to 66 kDa, 29 kDa, 12.4 kDa, 1.35 KDa are marked by inverted triangles labelled A, B, C, and D, respectively.

To examine whether the four DNA-recognition sites identified in the PIF dataset (H348, E352, R355, and R356) evolved conservatively, we screened the MYC-bHLH genes of *Marchantia polymorpha* (MapolyY_B0018.1 and Mapoly0018s0018.1), *Physcomitrium patens* (Pp3c3_9970V3.1, Pp3c11_15370V3.1, and Pp3c13_11550V3.1), *Zea mays* (Zm00001d030028_T001, Zm00001d047017, Zm00001d043706_T001, and Zm00001d007536_T001), *Ananas comosus* (Aco018875.1, Aco010845.1, Aco005839.1, and Aco024108.1) and *Arabidopsis thaliana* (AT4G17880.1, AT5G46760.1, AT2G46510.1, and AT1G32640) in Phytozome (https://phytozome-next.jgi.doe.gov/). The alignment was conducted via the MUSCLE algorithm, which was implemented in MEGA6. The four amino acid residues exhibited an across-gene conservation pattern (red arrows in Supplemental Figure 4).


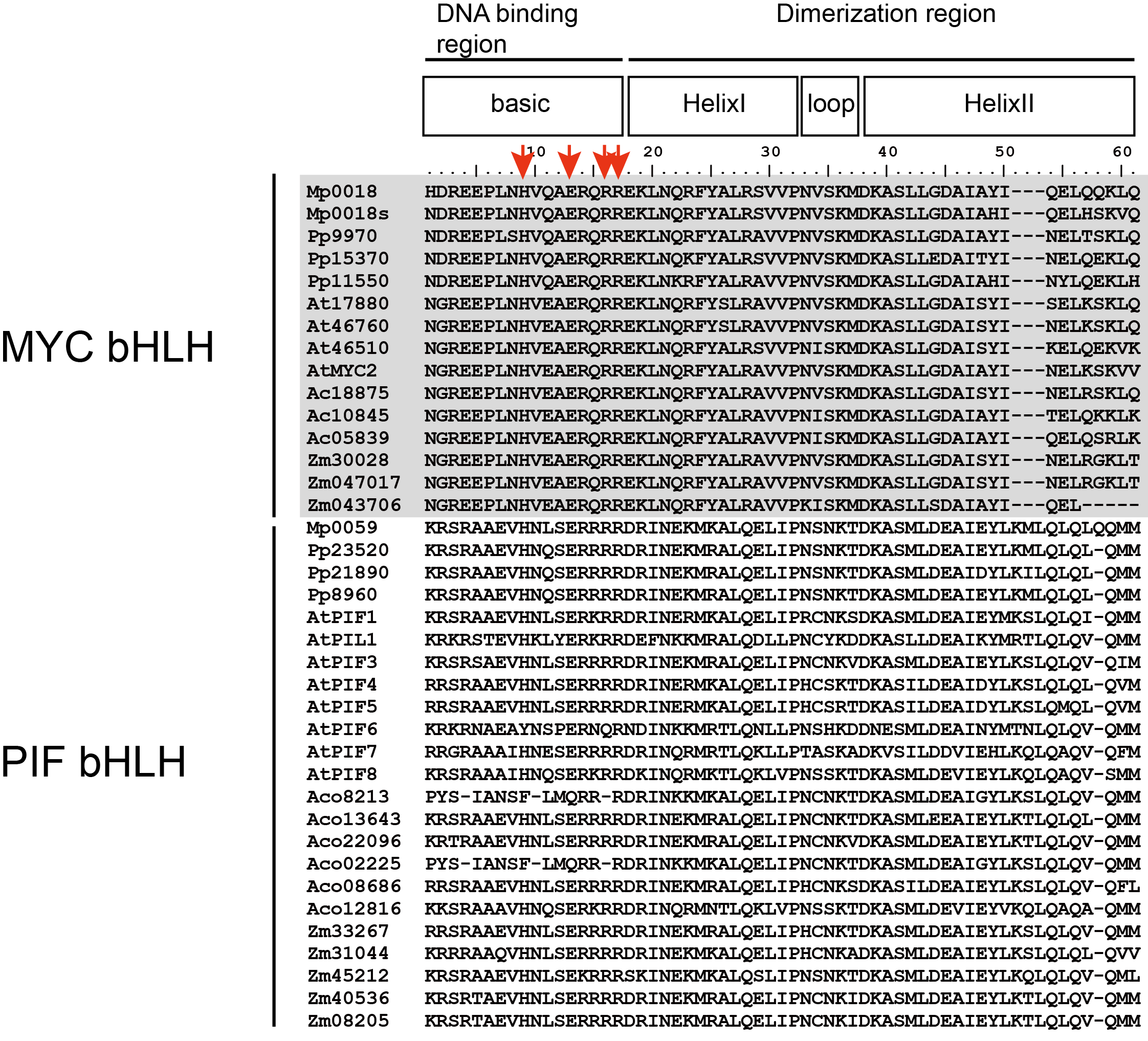


**Supplemental Figure 4**. The four conserved amino acid residues for DNA recognition inferred from MYC- and PIF-bHLH alignment across embryophytes, indicated by the red arrows.

To clarify the G-box and E-box distribution pattern among AtPIF3-bHLH targeting genes upstream, we selected 49 genes interacting with AtPIF3 in the light-signaling pathway, following Soy *et al.* (2016). These genes were submitted to KAAS, an automatic annotation server, and implemented in KEGG for functional assignment. The 49 genes were classified into five classes: metabolic pathways (6); biosynthesis of secondary metabolites (2); genetic information processing (4); environmental adaption (4); and unclassified (37; Supplemental Figure 5).

**Supplemental Figure 5**. Functional categorization of the AtPIF3-interacting gene set. Functional classification of these 49 genes was conducted by applying the single-directional best hit (SBH) method implemented in KAAS.

The upstream region (2000 bp) of each target was retrieved from the NCBI database (<https://www.ncbi.nlm.nih.gov/genome/gdv?org=arabidopsis-thaliana>). The promoter region of each gene was submitted to the PlantPan 4.0 web interface for G-box and E- box identification (<http://plantpan.itps.ncku.edu.tw/plantpan4/promoter_analysis.php>). Genes containing G-box were found to outnumber those without G-box (Supplemental Figure 4), suggesting that G-box is dominant in light-signaling pathways. This G-box dominance may hint at the reason for the binding specificity of AtPIF3-bHLH, especially when it encounters multiple types of E-box in a promoter region.

**Supplemental Figure 6**. Summary table of light-signaling pathway genes with and without G-box. The number of genes containing G-box is 3, 1, 2, 2, and 28 in the metabolic pathways, biosynthesis and secondary metabolites, genetic information processing, environmental adaptation, and unclassified groups, respectively. The number of genes without G-box is 3, 1, 2, 2 and 9, respectively.

To evaluate whether the AtPIF3-bHLH exhibits a preference for the seventh flanking-region nucleotide of G-box with an asymmetric binding mode in our modeling, we identified the seventh nucleotide in each identified G-box. G-box sequences with a G or a T in the seventh position (5′-CACGTGG-3′ and 5′-CACGTGT-3’) were the most common, followed by A and then C. These results suggest that AtPIF3-bHLH exhibits a preference for guanine and thymine at this location.

**Supplemental Figure 7**. Composition of identified G-box sequences in terms of their seventh nucleotides. There were 24 with G in this position, 21 with T, 15 with A, and four with C.

The core region of E-box allows for any nucleotide substitution (5′-CANNGC-3′), and its common sequences in light-signaling pathways have until now been unknown. A CG in the sequence occurred most frequently (71 times), followed by TT, then CA, then AA, and finally GT. The occurrence of core region types may be connected to the binding preferences identified in our competitive fluorescence-based electrophoretic mobility shift assay (fEMSA; Figure 7C). First, AtPIF3-bHLH exhibited specific binding to G-box, suggesting that G-box is favored. The residue signal we observed in PBE-box (5′-CACATG-3′) and E-box (5′-CAAATG-3′) together suggest a partial binding pattern between these two motifs. Lastly, the 5′-CAAGTG-3′ sequence exhibited weak AtPIF3-bHLH binding ability, according to our competitive fEMSA. Considering the binding abilities identified in that assay and the frequency of core region types in light-signaling-pathway genes, we concluded that AtPIF3-bHLH exhibits a frequency-based preference for E-box. This selection scenario could be described as “a combination of a higher frequency of encounters and a stronger binding preference”.

**Supplemental Figure 8**. The frequency distribution of E-box core region compositions in 49 light-signaling-pathway genes.

The location of every identified G-box and E-box is shown below (Supplemental Figures 7–10). Genes containing G-box are labelled with purple circles. Genes without G-box are labelled with a star. In addition, the seventh nucleotides of G-box and E-box are indicated in red.


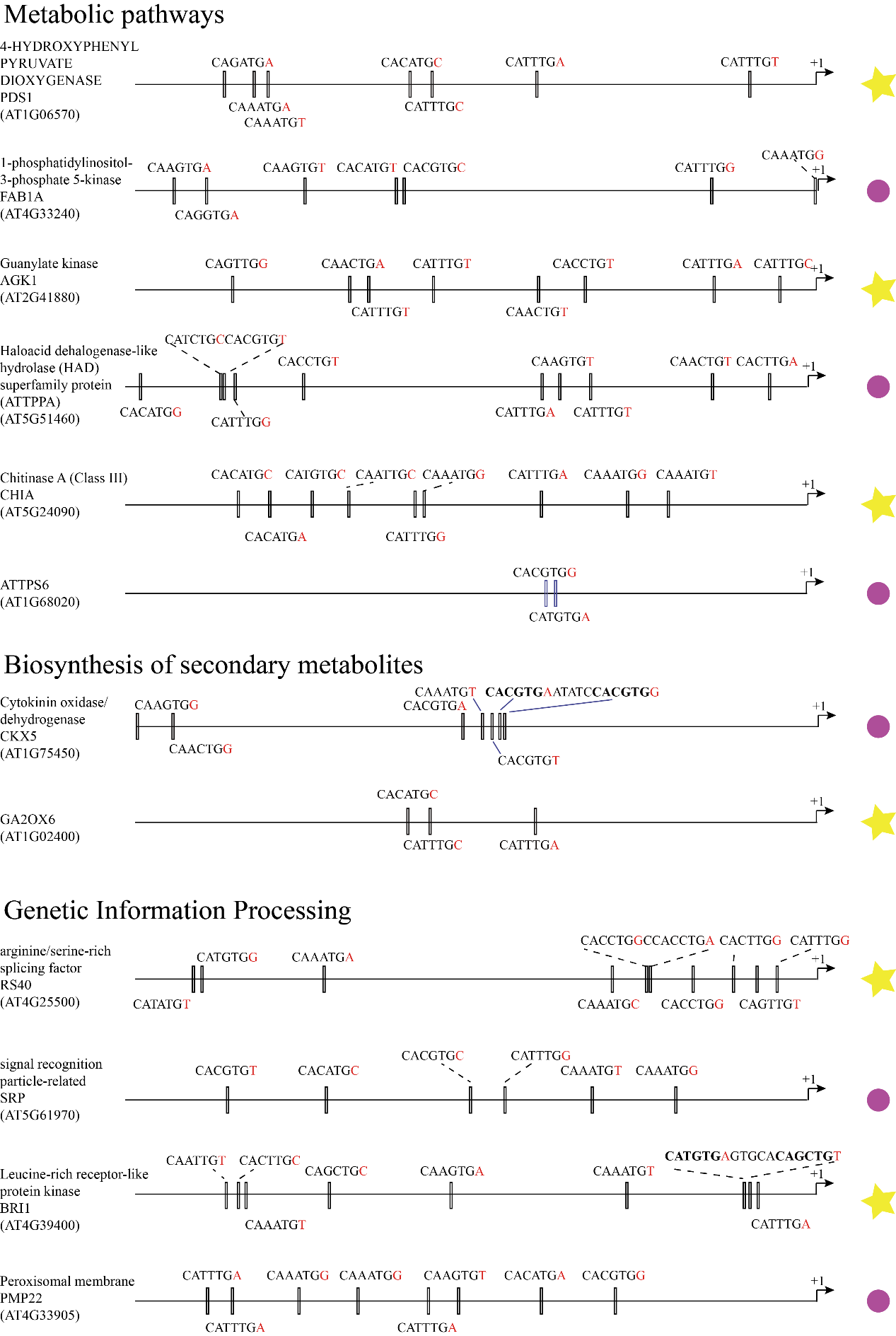


**Supplemental Figure 9**. G-box and E-box locations in sequences retrieved from genes in the metabolic pathways, biosynthesis processes of secondary metabolites, and genetic information processing groups.


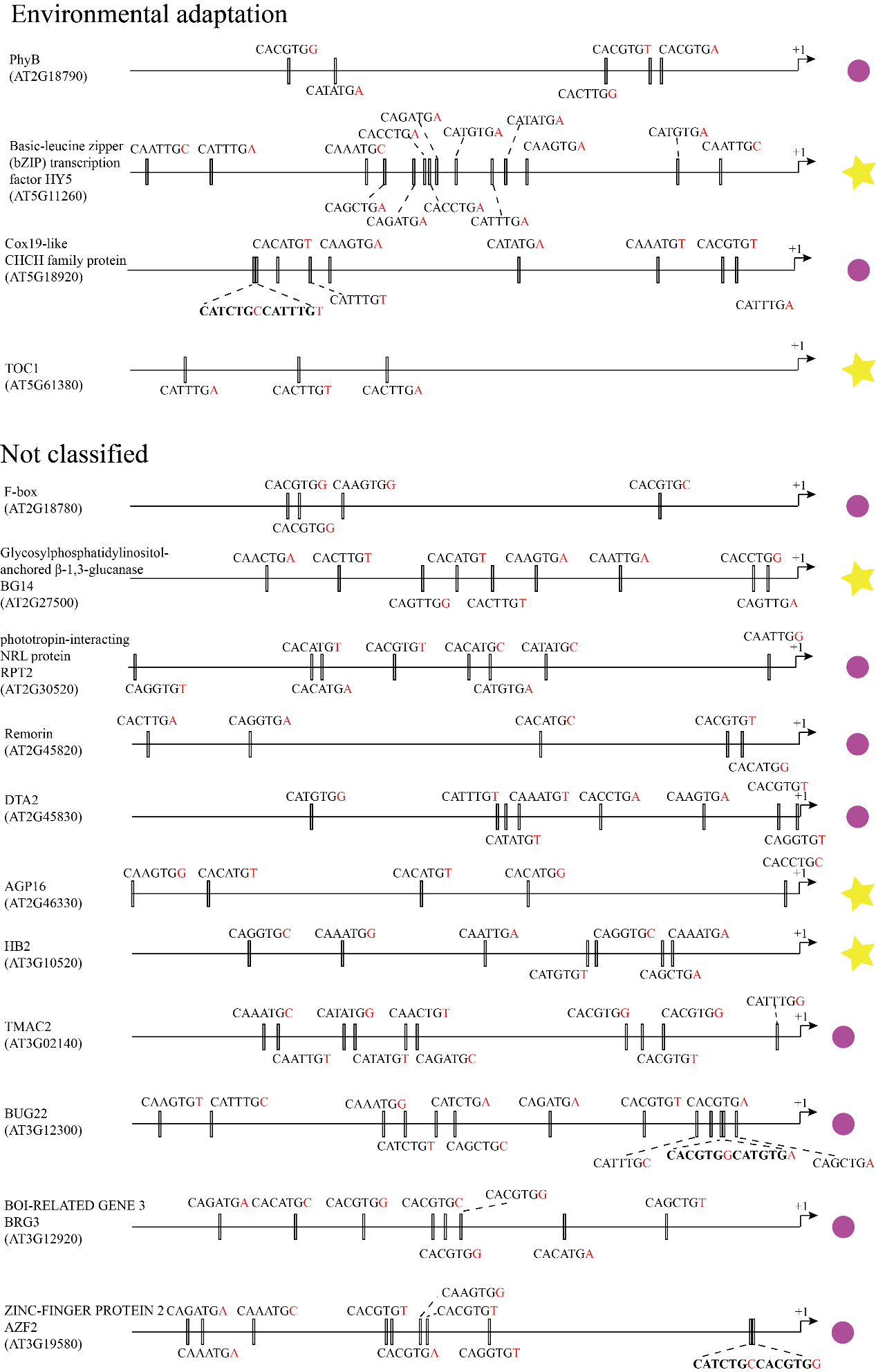


**Supplemental Figure 10**. G-box and E-box locations in genes in the environmental adaptation group and 11 of the unclassified genes.


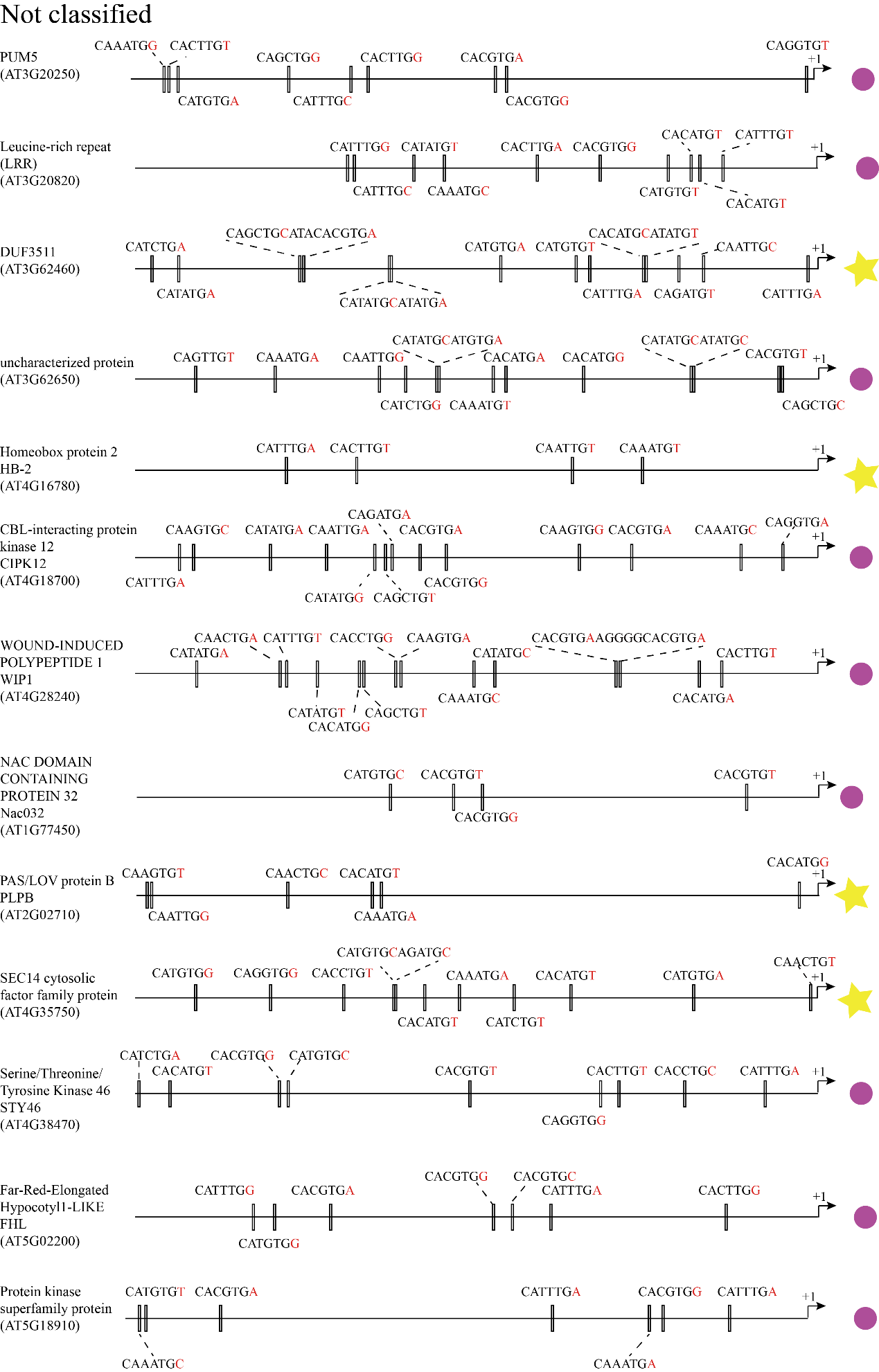


**Supplemental Figure 11**. G-box and E-box locations in 13 of the unclassified genes.


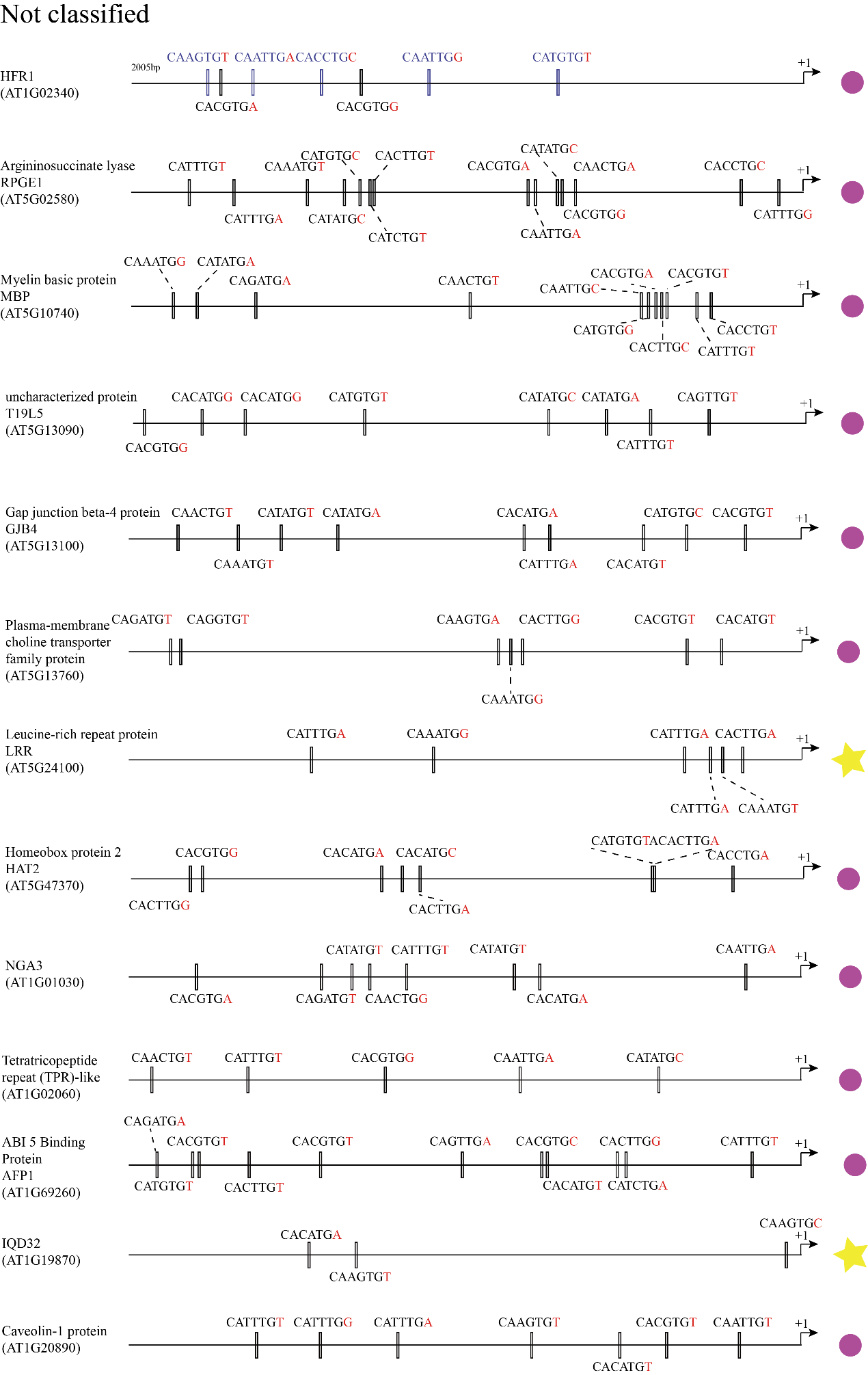


**Supplemental Figure 12**. G-box and E-box locations in 13 of the unclassified genes.

**
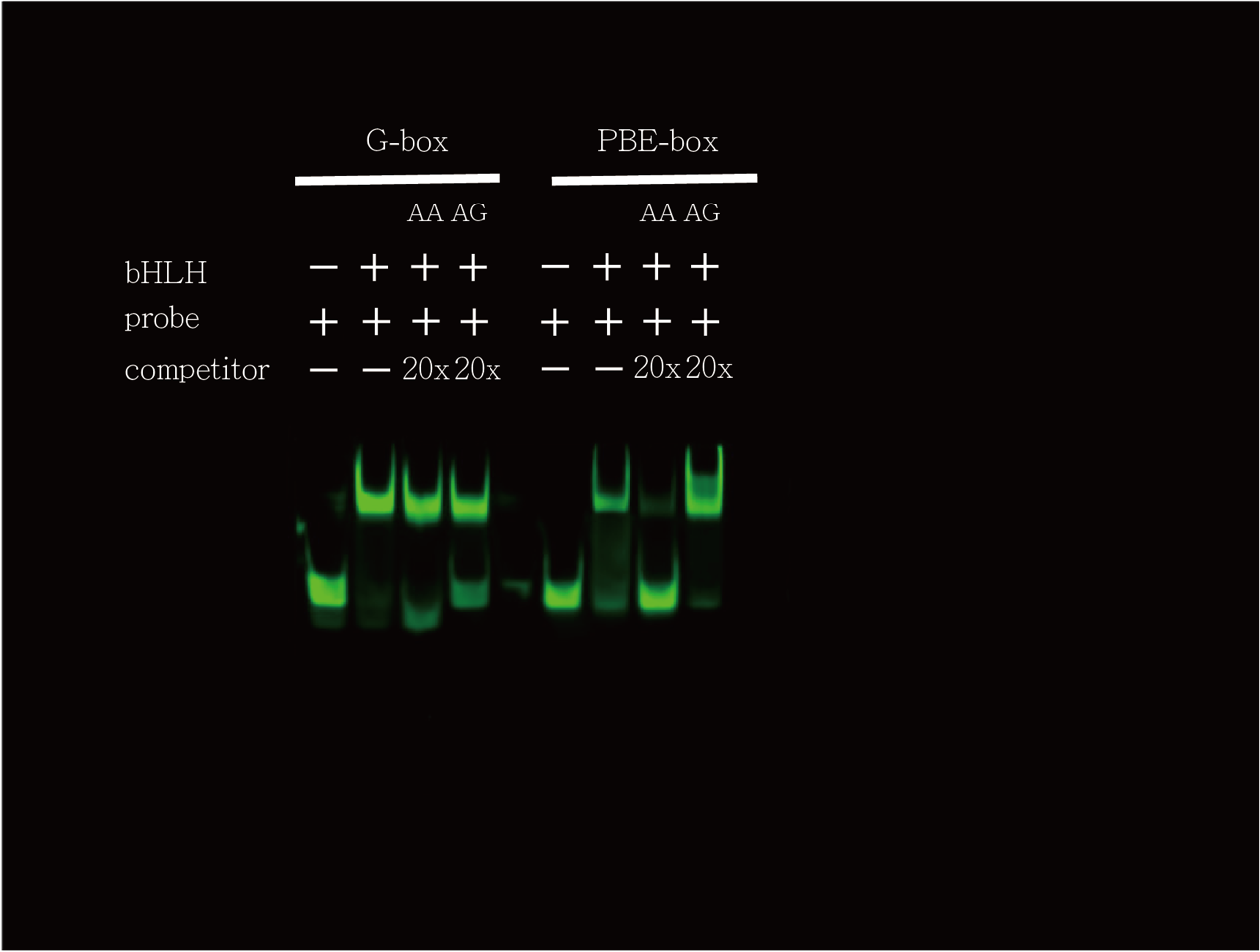
**

**Supplemental Figure 13.** The original SDS-PAGE gel of Figure 7C.


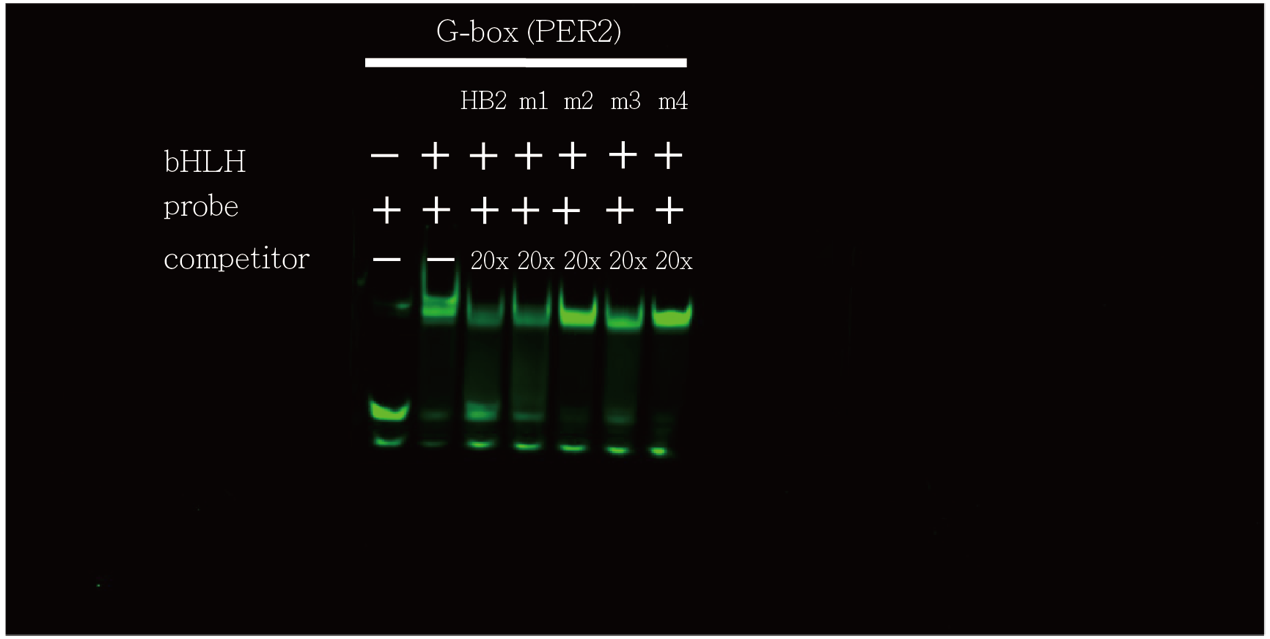


**Supplemental Figure 14.** The original SDS-PAGE gel of Figure 7D.


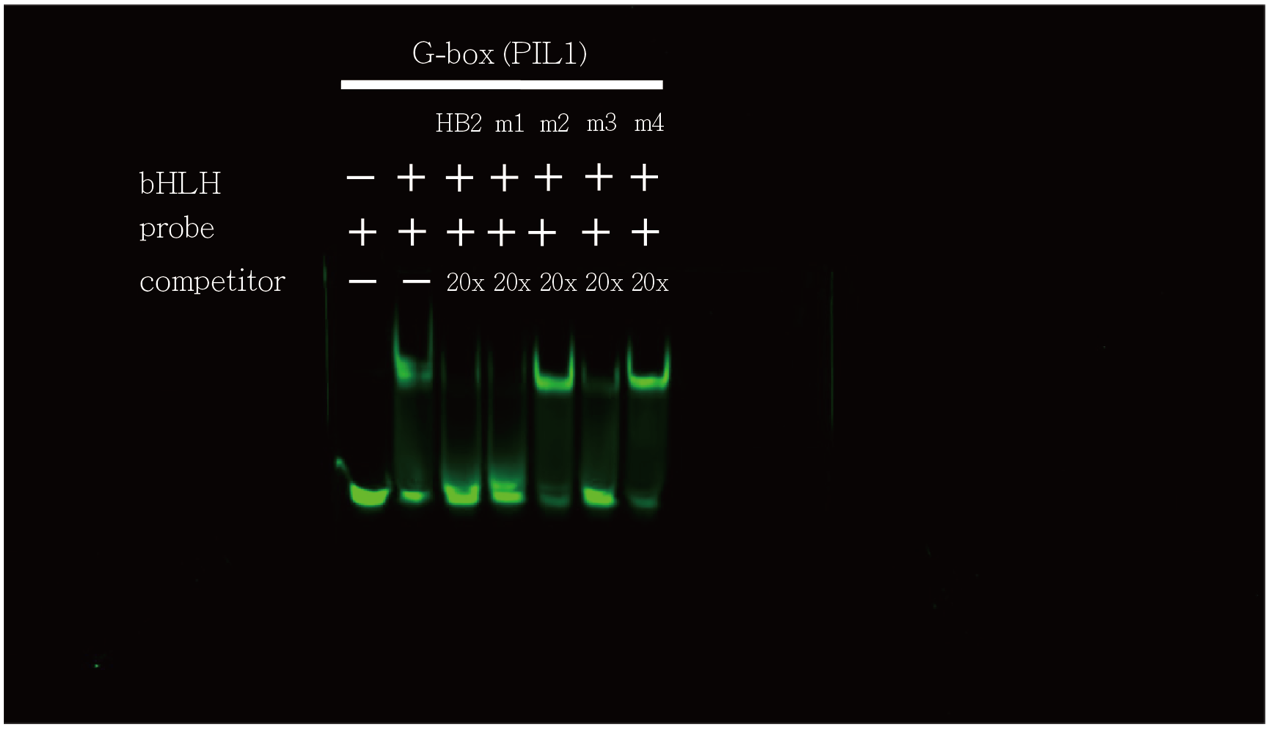


**Supplemental Figure 15.** The original SDS-PAGE gel of Figure 7E.
